# Global distribution, climatic preferences and photosynthesis‐related traits of C_4_ eudicots and how they differ from those of C_4_ grasses

**DOI:** 10.1002/ece3.10720

**Published:** 2023-11-12

**Authors:** Jessica A. Berasategui, Anže Žerdoner Čalasan, Alexander Zizka, Gudrun Kadereit

**Affiliations:** ^1^ Prinzessin Therese von Bayern Lehrstuhl für Systematik, Biodiversität & Evolution der Pflanzen Ludwig‐Maximilians Universität München München Germany; ^2^ Institute for Molecular Physiology Johannes Gutenberg‐University Mainz Mainz Germany; ^3^ Department of Biology Philipps‐University Marburg Marburg Germany; ^4^ Botanischer Garten München‐Nymphenburg und Botanische Staatssammlung München Staatliche Naturwissenschaftliche Sammlungen Bayerns München Germany

**Keywords:** biome, C_4_ photosynthesis, climatic preferences, desert, GBIF, salt tolerance, succulence

## Abstract

C₄ is one of three known photosynthetic processes of carbon fixation in flowering plants. It evolved independently more than 61 times in multiple angiosperm lineages and consists of a series of anatomical and biochemical modifications to the ancestral C_3_ pathway increasing plant productivity under warm and light‐rich conditions. The C_4_ lineages of eudicots belong to seven orders and 15 families, are phylogenetically less constrained than those of monocots and entail an enormous structural and ecological diversity. Eudicot C_4_ lineages likely evolved the C_4_ syndrome along different evolutionary paths. Therefore, a better understanding of this diversity is key to understanding the evolution of this complex trait as a whole. By compiling 1207 recognised C_4_ eudicots species described in the literature and presenting trait data among these species, we identify global centres of species richness and of high phylogenetic diversity. Furthermore, we discuss climatic preferences in the context of plant functional traits. We identify two hotspots of C_4_ eudicot diversity: arid regions of Mexico/Southern United States and Australia, which show a similarly high number of different C_4_ eudicot genera but differ in the number of C_4_ lineages that evolved in situ. Further eudicot C_4_ hotspots with many different families and genera are in South Africa, West Africa, Patagonia, Central Asia and the Mediterranean. In general, C_4_ eudicots are diverse in deserts and xeric shrublands, tropical and subtropical grasslands, savannas and shrublands. We found C_4_ eudicots to occur in areas with less annual precipitation than C_4_ grasses which can be explained by frequently associated adaptations to drought stress such as among others succulence and salt tolerance. The data indicate that C_4_ eudicot lineages utilising the NAD‐ME decarboxylating enzyme grow in drier areas than those using the NADP‐ME decarboxylating enzyme indicating biochemical restrictions of the later system in higher temperatures. We conclude that in most eudicot lineages, C_4_ evolved in ancestrally already drought‐adapted clades and enabled these to further spread in these habitats and colonise even drier areas.

## Graphical abstract

We identified global centres of species richness for C_4_ eudicots and C_4_ grasses and discussed the climatic preferences in relation to plant functional traits. C_4_ eudicots were abundant in deserts and xeric shrublands, tropical and subtropical grasslands, savannas and shrublands. We found C_4_ eudicots to occur in areas with less annual precipitation than C_4_ grasses which can be explained by frequently associated adaptations to drought stress such as among others succulence and salt tolerance.

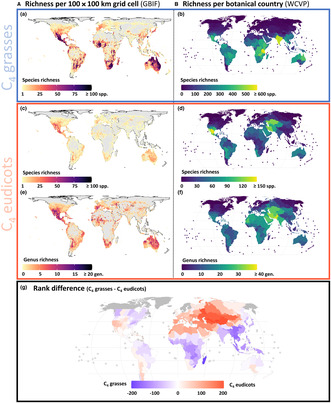

## 1 INTRODUCTION

By the early 1950s, it was widely assumed that all plants use the same C_3_ carbon fixation pathway, the Calvin‐Benson‐Bassham cycle (CBB‐cycle; Bassham et al., 1950). Shortly after a brief note on the discovery of a four‐carbon CO_2_ fixation pathway in sugarcane – now known as C_4_ photosynthesis – published in the 1954 Annual Report of the Hawaiian Sugar Planters Association Experiment Station (Burr et al., 1957; Hatch, 2005), researchers set out to investigate this unexplored photosynthetic pathway. They found that the CBB cycle and RuBisCO were restricted to bundle sheath cells (BSC) and that in the mesophyll cells (MC) an auxiliary carbon fixing pathway with phosphoenolpyruvate carboxylase (PEPC) as the key enzyme generated C_4_ molecules that are transported into the BSC and fuel the CBB‐cycle. Since its discovery, understanding C_4_ photosynthesis has become a vibrant research discipline, integrating the fields of biochemistry, physiology, organismic biology, ecology and evolution (Langdale, 2011). Evolving knowledge about the C_4_ pathways has been published in various reviews (see Furbank and Kelly, 2021; Niklaus and Kelly, 2019; Sage et al., 2018; Schlüter and Weber, 2020 for four recent ones on different aspects of C_4_ photosynthesis) and special issues (e.g., JXB special issue: C_4_ Photosynthesis – 50 years of discovery and innovation – Von Caemmerer et al., 2017).

**FIGURE 1:**
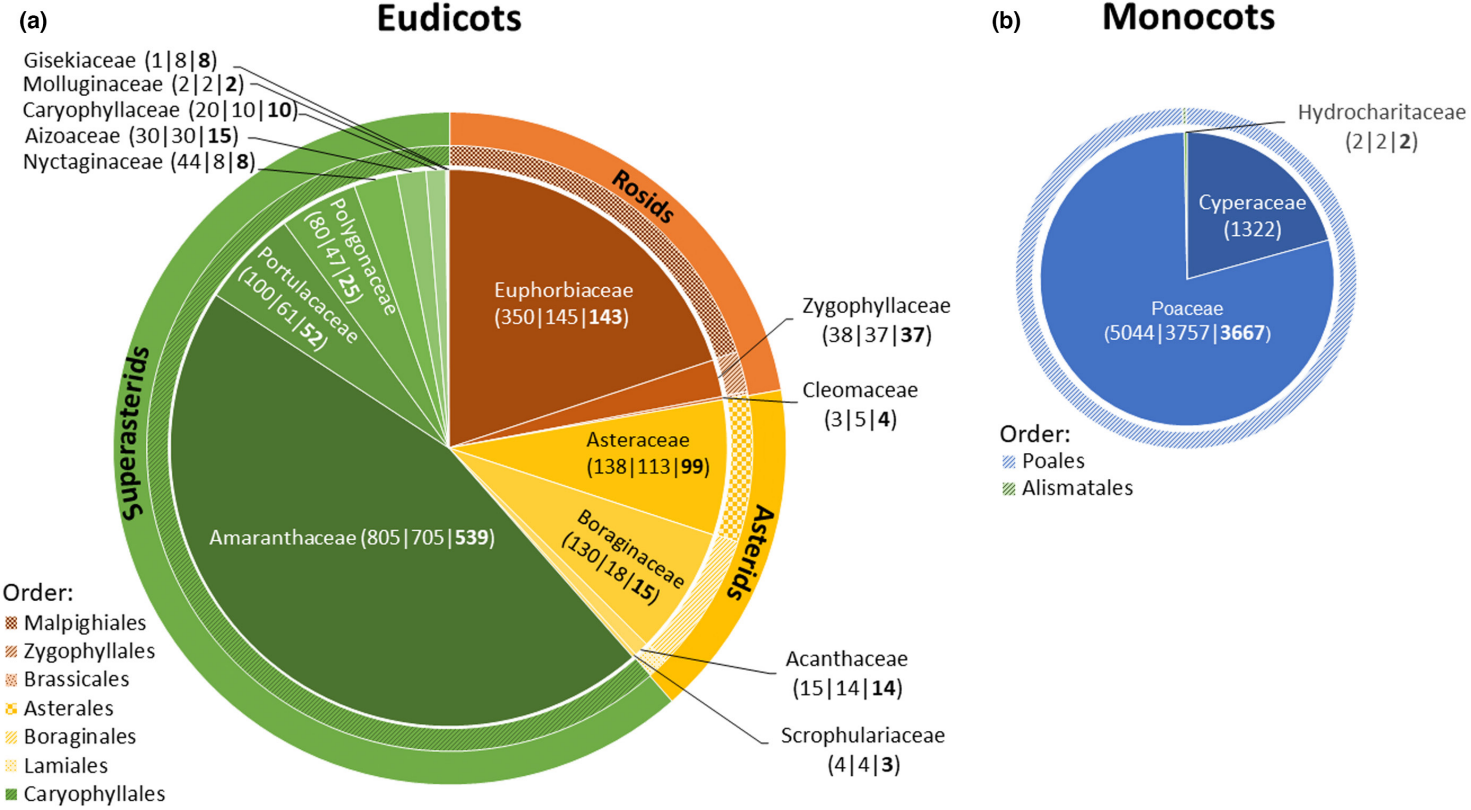
Angiosperm families in eudicots (a) and monocots (b) including C_4_ species. The first number represents the total number of C_4_ species per family according to Sage (2017) the second number represents the number of C_4_ species with verified information about C_4_ photosynthesis performance (Table S1) the number in bold shows the remaining number of C_4_ species after various data cleaning steps (Table 1). Proportions shown in the diagrams and mentioned in the text were calculated according to species numbers in Sage (2017) but do not change much when based on the other two reduced numbers.

C_4_ photosynthesis evolved in at least 18 angiosperm families and more than 60 times independently (Sage, 2017; Sage et al., 2018; Figure 1a). Around 80% of the C_4_ species are found in the Poales with 5044 C_4_ species in Poaceae and 1322 C_4_ species in Cyperaceae. With two C_4_ species in Hydrocharitaceae (Alismatales), this adds up to 6368 C_4_ species in 339 genera in monocots, opposed to around 1777 eudicot C_4_ species in 79 genera (Sage, 2017 and ref. therein) (Figure 1b). Interestingly, the eudicot C_4_ lineages are phylogenetically more equally distributed and occur in three rosid and four asterid families belonging to six different orders. Nevertheless, the C_4_ species‐richest eudicot clades are restricted mostly to eight families of the Caryophyllales (Figure 1a, based on Sage, 2017: table 3). Amaranthaceae s.l. (Caryophyllales) is by far the most species‐rich C_4_ eudicot family, followed by Euphorbiaceae (Malpighiales, rosids), Asteraceae and Boraginaceae (Asterales and Lamiales, respectively, asterids; Figure 1a). Although C_4_ photosynthesis in eudicots is phylogenetically more widespread and ecologically and structurally more diverse than in monocots (e.g., Muhaidat et al., 2007; Rudov et al., 2020), the latter have received more attention (mainly in the Poaceae). This is partly due to C_4_ grasses, such as maize and sugarcane, being initial model species of C_4_ research, thus making their close relatives the focus of C_4_ research even today (Hatch, 2005). Furthermore, research focus on grasses can be attributed to their great economic and ecological importance (Linder et al., 2018). However, understanding the diversity of the C_4_ syndrome in eudicots is key to understand the evolution of this complex trait (Heyduk et al., 2019) because C_4_ eudicot lineages evolved the C_4_ syndromes along different evolutionary paths (e.g., Bohley et al., 2015; Kadereit et al., 2012; Lauterbach et al., 2019).

C_4_ photosynthesis, which includes an auxiliary pathway to reduce photorespiration, likely arose in hot, dry and/or saline regions where C_3_ photosynthesis performance is reduced (Sage et al., 2018). This is achieved by the fixative enzyme, phosphoenolpyruvate‐carboxylase (PEPC), and by generating a local high CO_2_ concentration around the key enzyme RuBisCO, to reduce its oxygenase activity (Sage et al., 2012). C_4_ photosynthesis is usually associated with warm habitats with high evapotranspiration. Yet, the distribution of C_4_ plants cannot be explained entirely by individual environmental factors (Christin & Osborne, 2014) because C_4_ species occur in a variety of habitats, for example on nutrient‐poor or fertile soils, in the tropics, in deserts or in the boreal zone, on open grasslands or forest undergrowth (Collins & Jones, 1986; Mahdavi & Bergmeier, 2018; Rudov et al., 2020). This diversity results from the multifaceted evolutionary history of the C_4_ pathway (Christin & Osborne, 2014; Sage et al., 2011, 2018). However, studies linking the evolution of adaptive traits and ecological niches in C_4_ lineages are still insufficient. Lundgren et al. (2015) for example showed that C_4_ photosynthesis does not initially lead to a shift of the ancestral niche in *Alloteropsis semialata* J. Presl (Poaceae), but rather expands its niche to cover a wider range of conditions that include the ancestral ones. This improves the success of occasional long‐range dispersal events and thus increases the geographical range (Lundgren et al., 2015). As C_4_ photosynthesis is a complex syndrome that increases the efficiency of plants to use available water and nitrogen, C_4_ might be advantageous under various environmental conditions, but evolved predominantly in the tropics and subtropics (Griffiths et al., 2013; Sage et al., 2018).

### 1.1 Global expansion of C_4_ grasses

C_4_ plants account for one‐quarter of the earth's primary terrestrial production, and almost a quarter of the Earth's surface is dominated by C_4_ grasslands and savannas (Barbehenn et al., 2004; Grace et al., 2006; Sage et al., 2018). C_4_ grasses likely intruded into C_3_ grasslands and forests from open biomes of warm regions, subsequently replaced them during the late Miocene to the Pliocene (3–8 Mya) and expanded worldwide into drier biomes (Edwards & Smith, 2010; Ehleringer et al., 1997; Osborne & Freckleton, 2009). However, molecular research suggested that C_4_ photosynthesis in several grass lineages evolved earlier, around 18–30 Mya (mid/late‐Oligocene) presumably in warm, arid locations where water limitation was the main selective force to increase photorespiration (Christin et al., 2011; Zhou et al., 2018). As the atmospheric CO_2_ in the late Miocene fell below ~300 ppm (Royer, 2006), a small number of hyperdominant C_4_ grass species that were able to outcompete C_3_ and C_4_ relatives became dominant due to their advantage of a low CO_2_ compensation point (Christin & Osborne, 2014; Lehmann et al., 2019). South America seems to be the major hotspot for the origin of C_4_ grasses (Sage et al., 2011), and today, C_4_ grasses are confined mostly to tropical and subtropical areas (Shoko et al., 2016; Woodward et al., 2004; Woodward & Lomas, 2004). Climatic patterns and the distribution of C_4_ grasses in North America suggest that high minimum temperatures during the growing season favour C_4_ grasses at regional scale (Teeri & Stowe, 1976). Yet, at the local scale, topographic and edaphic variables may exert more influence (Yan & de Beurs, 2016).

### 1.2 Evolutionary and ecological diversity of C_4_ photosynthesis in eudicots

Individual C_4_ lineages had originated independently from the Oligocene into the Quaternary (Christin et al., 2011; Niklaus & Kelly, 2019). In Amaranthaceae s.l., which includes the largest number of C_4_ lineages in eudicots, the earliest assumed origins of C_4_ date back to the Oligocene and are roughly as old as the oldest C_4_ grass subfamily Chloridoideae, which evolved around 32–25 Mya. This implies that C_4_ eudicots are per se not younger than C_4_ monocots (Christin et al., 2008, 2011; Kadereit et al., 2012). However, many C_4_ lineages within eudicots (as well as in the monocots) originated in the Miocene when the climate got increasingly drier (Kadereit et al., 2003, 2010). In addition, there are many evolutionary young C_4_ lineages in eudicots, for instance in *Flaveria* (Asteraceae), *Sesuvium* (Aizoaceae) and *Tecticornia* (Chenopodiaceae s.s.) that arose approximately 5–1 Mya (Christin et al., 2011; Kadereit et al., 2012; Sage et al., 2012). Since the range size of C_4_ lineages as well as the physiological refinement of the C_4_ syndrome is highly dependent on time, the age of the respective C_4_ lineage needs to be taken into account when lineages are compared to each other (Niklaus & Kelly, 2019).

Although South America seems to be the hotspot of origins of the nowadays cosmopolitan C_4_ grasses, six geographic regions were highlighted as potential ancestral areas for C_4_ eudicot lineages. For most C_4_ eudicot lineages Central Asia, North America, South Africa, northeast Africa and Arabia count as centres of origin (Kadereit & Freitag, 2011; Sage, 2016; Sage et al., 2011), an assessment based mainly on current distribution that still awaits the review of detailed phylogenetic, biogeographical studies in the individual eudicot lineages (e.g., Lauterbach et al., 2019). Due to the diverse nature of C_4_ eudicots, no list of the global distribution of C_4_ eudicots has been compiled thus far.

### 1.3 Functional plant traits in C_4_ eudicots

Several traits such as succulence, salt tolerance, fast seed germination and longevity seem to be associated with C_4_ photosynthesis in eudicots (e.g., Kadereit et al., 2012, 2017). While succulence is generally rare among grasses (only in *Spinifex littoreus* (Burm.f.) Merr.; Ho et al., 2019), this trait results in an unmatched leaf and stem anatomical diversity in C_4_ eudicots (Bohley et al., 2015; Kadereit et al., 2003; Muhaidat et al., 2007, 2018; Voznesenskaya et al., 2017). Grasses usually show a classical Kranz anatomy (similar to an atriplicoid leaf anatomy), whereas eudicots exhibit a broad variety of succulent and non‐succulent C_4_ leaf types in addition to the atriplicoid type (Edwards & Voznesenskaya, 2011). Among the succulent C_4_ leaf types, the annual *Suaeda aralocaspica* (Bunge) Freitag (= *Borszczowia aralocaspica*; Amaranthaceae s.l.), *Bienertia cycloptera* Bunge ex Boiss, *B. sinuspersici* Akhani and *B. kavirense* Akhani are particularly noteworthy because their C_4_ photosynthesis is carried out within a single photosynthetic cell and without the supposedly mandatory C_4_ Kranz anatomy (Akhani et al., 2005, 2012; Freitag & Stichler, 2000; Sharpe et al., 2020; Voznesenskaya et al., 2002, 2003). Although many different examples of anatomical diversity in C_4_ eudicots have been found, there are still many representatives of the other C_4_ eudicot lineages that are not well characterised and might contribute to the ecological and morphological diversity (Muhaidat et al., 2007).

Most succulent C_4_ species tolerate elevated salinity, suggesting that their succulence is primarily an evolutionary response to (physiological) drought. While in grasses a repeated gain and loss of salt tolerance throughout the history of the family prevails and halophytic grass species are isolated at the tips of the phylogeny (Bromham & Bennett, 2014), there are multiple evolutionary older halophytic lineages among eudicots that additionally acquired C_4_ photosynthesis. This is particularly the case for C_4_ lineages of Amaranthaceae (Kadereit et al., 2012, 2017; Piirainen et al., 2017) but also for Gisekiaceae (Bissinger et al., 2014), Sesuvioideae‐Aizoaceae (Bohley et al., 2015) and Euphorbiaceae (Ghazanfar et al., 2014; Rudov et al., 2020). Some eudicot lineages acquired even further alternative carbon fixation pathways. The widespread succulent annual *Portulaca oleracea* L. is a halophytic C_4_ species that is able to conduct both C_4_ and CAM photosynthesis depending on the environmental conditions (Ferrari et al., 2020, 2022).

While C_4_ trees and large shrubs are generally rare, several eudicot C_4_ species can be woody and/or perennial such as *Anabasis* from Eurasian steppes and semi‐deserts (Lauterbach et al., 2019); the saxaul (*Haloxylon ammodendron* (C.A. Mey.) Bunge ex Fenzl), which dominates continental deserts of Asia (Pyankov et al., 1999) and the Hawaiian C_4_ trees *Euphorbia olowaluana* Sherff and *E. herbstii* (W.L. Wagner) Oudejans (Pearcy & Troughton, 1975; Young et al., 2020).

Despite anatomical and ecological differences, the biochemical forms that exist in C_4_ photosynthesis are similar in grasses and eudicots. There are three biochemical subtypes in both grasses and eudicots, which are usually constant in a C_4_ lineage, but may vary within and between plant families: NADP‐malic enzyme (ME; e.g., in Caryophyllaceae; Sage et al., 2011), NAD‐ME (e.g., C_4_ species in Boraginaceae, Cleomaceae; Muhaidat et al., 2007), and the third further decarboxylating enzyme, PEP‐CK that is more common in C_4_ monocots (Wang et al., 2014). Due to the high number of fast growing and highly productive C_4_ grasses many of which are interesting biofuel crops and phytoremediation plants (such as *Miscanthus*; Pidlisnyuk et al., 2014), one might assume that C_4_ grasses are more competitive than C_4_ eudicots given the right growing conditions. However, interestingly the species with the fastest CO_2_ assimilation rates of 80 μmol m^−2^ s^−1^ at 325 μmol mol^−1^ is not a grass species but *Amaranthus palmeri* S. Watson (Amaranthaceae, Ehleringer, 1983; Sage, 2017).

### 1.4 Scope and aims

C_4_ grasses entail the majority of C_4_ species and dominate in biomass production, yet the anatomical, physiological and ecological diversity of C_4_ syndromes seems larger in C_4_ eudicots. While shifts to C_4_ physiology in grasses probably represent a pre‐adaptation to open and arid subtropical habitats (Edwards & Smith, 2010; Osborne & Freckleton, 2009), the evolution of the C_4_ pathway in eudicots, e.g., Amaranthaceae s.l. (incl. Chenopodiaceae), Nyctaginaceae and Sesuvioideae, is more likely a post‐adaptation to the selection pressure in dry, saline and coastal environments that enabled survival in these habitats (Bohley et al., 2015; Kadereit et al., 2012; Khoshravesh et al., 2020). Already, Stowe and Teeri (1978) suggested that C_4_ eudicots do not follow the climate preferences that have been reported for C_4_ grasses and therefore might have followed a different evolutionary pathway to C_4_.

In this study, we aimed to characterise the global occurrence of C_4_ eudicots, identify diversity hotspots and climatic preferences, and assign these to functional traits such as succulence, salt tolerance, biochemical subtype and anatomical leaf type. We hypothesised that the phylogenetic and structural diversity of C_4_ eudicots is reflected in their colonisation of a wide range of climatic regions and environments and that the combination of C_4_ photosynthesis with other traits enabled C_4_ eudicots to invade areas not or less frequently colonised by C_4_ grasses. To test this hypothesis, we compare the biogeographic patterns found in eudicots with those of the more species‐rich C_4_ grasses. Moreover, we link the geographical origins of C_4_ photosynthesis to the diversity hotspots discovered in those C_4_ eudicot lineages examined within a phylogenetic framework.

## 2 MATERIALS AND METHODS

We compiled an initial dataset of C_4_ eudicots according to Sage (2017). This list consisted of 16 eudicot families with indication of lineages and the number of C_4_ species per lineage (Table S1). In order to list each C_4_ species, literature research was conducted. If trait data were available, we recorded leaf anatomy, biochemical subtypes (NAD‐ME, NADP‐ME), succulence, woodiness, salt tolerance and life form (perennial, annual) from floras, revisions, reports, databases and online sources (see sources in Table S1). To reduce the artificial increase in species numbers and distribution areas due to synonymisation, we cross‐checked for synonyms using plantsoftheworldonline.org (POWO, 2020). In grasses, there are over 60,000 published scientific names corresponding to approximately 11,313 accepted species (Clayton et al., 2002 onwards; Osborne et al., 2014). Our list of C_4_ eudicot species includes members of 15 families (Chenopodiaceae included in Amaranthaceae) with 1207 accepted species and a total of around 3969 synonyms. These 1207 species have verified information about C_4_ photosynthesis performance and the literature and/or online resources provided detailed information about the traits discussed above. For comparison with C_4_ grasses, we compiled a list of 309 genera following Osborne et al. (2014) (Table S1), as all species of these genera are assumed to perform C_4_ photosynthesis.

### 2.1 Occurrence data

We extracted georeferenced occurrence data of the 1207 C_4_ eudicot species from the Global Biodiversity Information Facility (gbif.org) using the “rgbif” v3.2.0 package (Chamberlain et al., 2020) in R (for DOIs see GBIF‐References; GBIF.org, 2020a, 2020b, 2020c, 2020d, 2020e, 2020f, 2020g, 2020h, 2020i, 2020j, 2020k, 2020l, 2020m, 2020n, 2020o). We downloaded occurrence records at species level for C_4_ eudicots (categorised by family) and at genus level for C_4_ grasses (categorised by family/subfamily) due to the aforementioned assumption (GBIF.org, 2020p, 2020q, 2021r, 2020s, 2020t). Records describing fossilised specimens, records based on literature alone and records of unknown origin were excluded (R code available at https://docs.ropensci.org/rgbif/articles/rgbif.html). GBIF records originated from a variety of sources, including human and machine observation (e.g., photograph), living and preserved specimens. For 208 C_4_ eudicot species (17%) included in our list of verified C_4_ species, no reliable occurrence data were available in GBIF.

### 2.2 Data cleaning

Since georeferenced occurrence records from public datasets such as gbif.org are error‐prone (Maldonado et al., 2015; Zizka et al., 2020), automated data cleaning of the C_4_ eudicot and C_4_ grasses coordinate datasets was performed with the “CoordinateCleaner” v2.0‐18 package in R (Zizka et al., 2019) using the default options. Following the process outlined in Zizka et al. (2019), erroneous records within 1000 m of country and/or province centroids and within 10,000 m of countries' capitals, within urban areas, records with locations as zeros, identical values, near GBIF headquarters, near biodiversity institutions and records on an ocean surface were removed. In addition to the “CoordinateCleaner,” the dataset was manually checked for incorrect synonymisation relying on plantsoftheworldonline.org as the taxonomic backbones of the GBIF are not always following the currently accepted taxonomic treatments by “The International Plant Names Index” (IPNI, 2020) and “World Checklist of Vascular Plants” (WCVP, Govaerts et al., 2021). Besides, duplicated coordinates, based on species name and coordinates, were removed. Likewise, the taxonomic reliability using the distribution information of plantsoftheworldonline.org was checked and occurrence points considered incorrect based on their distribution outside the native ranges of species were excluded. As a result of all cleaning steps, the number of coordinates was reduced from 1,012,557 to 247,205 occurrence points.

The cleaning of 2,296,101 occurrence records of C_4_ grasses was also carried out with the “CoordinateCleaner” package. Additionally, the occurrence points outside the native ranges and duplicate coordinates per species were excluded. Manual cleaning of the incorrectly synonymised species was not carried out here, as we focussed on the genus level only.

### 2.3 Analyses

We used 100 × 100 km grid cells to infer geographic patterns of C_4_ species richness, with an equal area Behrmann projection. Species richness maps for each C_4_ eudicot family for the uncleaned and cleaned GBIF dataset were generated using the package “speciesgeocodeR” v2.0‐10 (Töpel et al., 2017; Figures S1–S15). Grids with species numbers were calculated using *RichnessGrid*. In addition to the individual species richness maps for each C_4_ eudicot family, total species richness maps for C_4_ eudicots and C_4_ grasses were created with the cleaned datasets. These maps provide information on the total distribution (showing outstanding regions of C_4_ species richness) of both groups. Grids showing richness above 50 species were defined to be C_4_ species hotspots of high diversity.

Since data obtained from GBIF may be biased by unequal sampling in different areas (Hughes et al., 2021; Zizka et al., 2021), we additionally obtained data on species occurrences on the world geographic scheme for recording plant species Level‐3 (TDWG, 2001) from the World Checklist of Vascular Plants (WCVP, Govaerts et al., 2021) as comparison. The WCVP contains a complete (to the best of current knowledge) list of all vascular plant species and hence is less biased by differences in sampling (but in exchange for lower spatial resolution and unequally sized sample areas; Antonelli et al., 2023; Schellenberger Costa et al., 2023). We obtained the distribution of C_4_ eudicots from WCVP using the rWCVP package (Brown et al., 2023). First, we matched the names of our species list with WCVP using the wcvp_match_names function, with subsequent manual resolution of multiple matches (Table S2) and then obtained all natural, present, not doubtful occurrences of these species. For C_4_ grasses, we first matched the list of our genera with WCVP (Table S2) with sub‐sequential manual resolution of all multiple matches and then obtained the natural, present and undoubtful distributions of all species in these genera.

**FIGURE 2:**
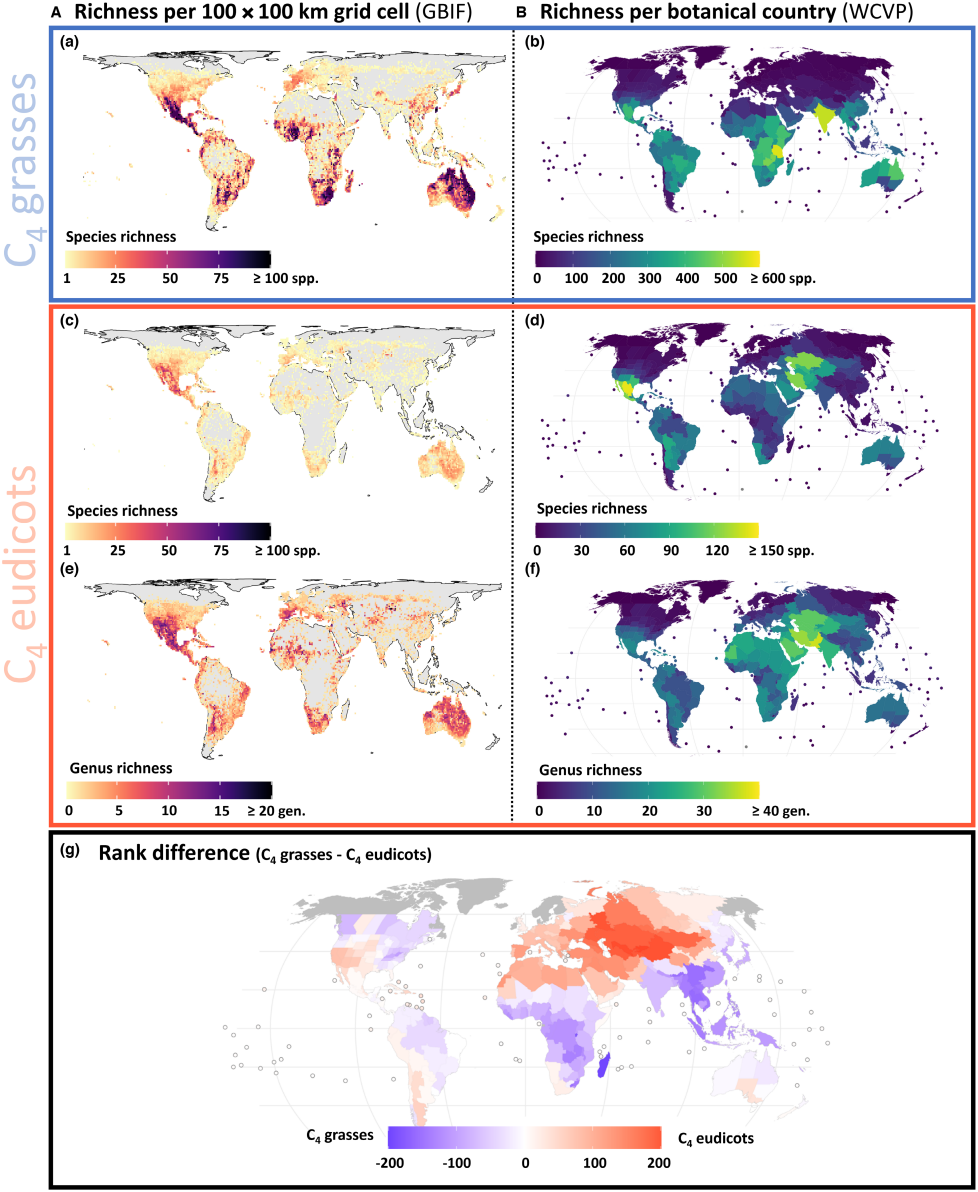
Global maps of total C_4_ grasses and C_4_ eudicots. A. Richness per 100 × 100 km grid cell. B. Richness per botanical country. (a) and (b) Species richness of C_4_ grasses. (c) and (d) Species richness of C_4_ eudicots. (e) and (f) Genus richness of C_4_ eudicots. (g) Rank difference (botanical countries ranked by the number of respective species) C_4_ grasses – C_4_ eudicots. Blue indicates botanical countries more important for C_4_ grasses, and red indicates botanical countries more important for C_4_ eudicots.

To compare the importance of individual regions between C_4_ eudicots and C_4_ grasses, we calculated the rank difference for each region, by first ranking each region by the number of C_4_ eudicot and C_4_ grass species (most species = 1, fewest species = 354) with a mean tie‐breaker (Figure 2g). We only included botanical countries with at least one C_4_ grass and at least one C_4_ eudicot.

Two bioclimatic variables (Bio1 – Annual Mean Temperature (°C*10); Bio12 – Annual Mean Precipitation (mm)) were extracted from WorldClim v.2 with a spatial resolution of 10 min (~340 km^2^) (Fick & Hijmans, 2017). We plotted annual mean temperature and precipitation values using ggplot2 to compare the distribution of C_4_ eudicots and C_4_ grasses along these two climatic variables (Wickham et al., 2016). Since the focus here is mainly on comparing these two groups and there is probably no systematic difference in bias between them, the cleaned GBIF dataset was used for this analysis. We are aware that this approach does not integrate biogeographical history which is beyond the scope of this paper. Likewise, boxplots for each family of C_4_ eudicots, the C_4_ grasses and all C_4_ eudicots together were calculated in relation to the climate variables by first calculating the mean values of each species for each climate variable. Statistical analyses were conducted in R v4.0.2 (R Core Team, 2020) using RStudio v1.2.5042 (RStudio, Inc., 2009–2020) and R Commander (Fox, 2005). We used a Kruskal–Wallis test (for between C_4_ eudicot families), followed by a post‐hoc Tukey–Kramer test to determine where the differences were, with a *p*‐value equal to or <.05 being considered statistically significant and a Mann–Whitney *U* test (between C_4_ eudicots and C_4_ grasses) to determine significance according to annual mean precipitation and annual mean temperature. A one‐way ANOVA test was performed to determine whether eudicot C_4_ species with NAD‐ME as the primary decarboxylating enzyme are distributed in areas with significantly lower annual precipitation than eudicot C_4_ species of the NADP‐ME subtype.

To display the distribution areas of each C_4_ eudicot family at the biome level (Olson et al., 2001), a table in Figure 4a was created. It shows the number of family species within a biome (Terrestrial ecoregions of the world; sensu Olson et al., 2001). Only plant families with the highest number of species in that study according to Figure 1a were selected for this table.

### 2.4 Literature survey

To place our findings on the diversity hotspots of C_4_ eudicots in a spatiotemporal framework, we conducted a literature review of phylogenetic and biogeographical studies incorporating C_4_ eudicot lineages in order to reveal the current understanding of the C_4_ photosynthesis origin in these groups. The aim was to assess whether a lineage developed C_4_ photosynthesis in a particular area (in situ origin) or developed C_4_ before colonising a particular area (ex situ origin).

## 3 RESULTS

### 3.1 The impact of data cleaning

We included only species for which direct evidence of C_4_ photosynthesis (such as C_4_‐like δ^13^C values or C_4_ leaf anatomy) is documented in the literature. This was the case for 1207 species of the approximately 1777 C_4_ eudicots according to Sage (2017). Since Sage (2017) estimates the number of C_4_ species per lineage, our refined C_4_ eudicot species list is substantially shorter (Table S1). For 208 of these 1207 C_4_ eudicot species, no occurrence points were documented in GBIF. Therefore, the final list of C_4_ eudicots analysed here included 999 species.

Performing the necessary cleaning steps reduced the raw C_4_ eudicot occurrence points dataset, which originally contained more than 1 million records, to less than a quarter (Table 1). After the use of the “CoordinateCleaner” package, 280,935 C_4_ eudicot records (27.75%) of 1,012,557 were removed (Table 1). A manual check for erroneous synonymisation removed 153,236 occurrence records from the remaining dataset. In the next step, 155,481 duplicate coordinates were removed. Notable is the additional reduction of 175,700 distribution points, after filtering out the outliers. After all these cleaning steps, we retained 963 species with a total of 247,205 occurrences. 620 species were represented by more than or equal to 10 records, whereas 343 species were represented by <10 records. Altogether, 75.59% of the occurrences for the C_4_ eudicots were excluded (Table 1).

**TABLE 1:** The impact of filtering on the raw occurrence datasets of the various eudicots plant families containing C_4_ species (including the number of occurrence points at each step). Beginning with the raw record list.

	Occurrences after downloading from GBIF^a^	Occurrences after applying CoordinateCleaner^b^	Occurrences after manual cleaning^c^	Occurrences after removing duplicates	Occurrences after removing outliers^d^
Acanthaceae	1380	1335	1335	988	855
Aizoaceae	4894	4009	4009	3135	2632
Amaranthaceae	645,813	463,332	336,492	242,485	144,100
Asteraceae	14,369	12,772	12,767	10,889	9562
Boraginaceae	5261	4782	4253	3156	3003
Caryophyllaceae	9338	8644	8606	6389	3224
Cleomaceae	2093	1735	1563	1163	925
Euphorbiaceae	122,123	85,559	82,052	61,509	45,285
Gisekiaceae	563	525	399	315	312
Molluginaceae	1347	1263	1240	1031	528
Nyctaginaceae	21,248	18,395	16,530	14,271	13,645
Polygonaceae	765	719	719	391	210
Portulacaceae	155,597	106,501	86,490	61,308	12,197
Scrophulariaceae	239	216	204	171	169
Zygophyllaceae	27,527	21,835	21,727	17,504	10,558
C4 eudicots	1,012,557	731,622	578,386	422,905	247,205

^a^ See references for DOI number.

^b^ CoordinateCleaner v2.0‐18 (Zizka et al., 2019).

^c^ Manual cleaning: Nomenclatural and taxonomic checking including [the correction of] wrong synonymisations.

^d^ Deleting outliers: after checking whether the native distribution information of plantsoftheworldonline.org matches the distribution country (Country Code) of each distribution point from GBIF.

In the raw C_4_ grasses dataset, which contained 2,296,101 distribution points for 271 C_4_ grass genera (GBIF.org, 2020p, 2020q, 2021r, 2020s, 2020t), around 382,595 points (16.66%) were removed after applying the “CoordinateCleaner” package. Excluding occurrence points outside the original distribution areas resulted in the elimination of 375,560 distribution points. Duplicate coordinates per species were removed, resulting in an additional 464,424 occurrence points being excluded. After all cleaning steps, approximately 53.25% distribution points were removed from the raw C_4_ grasses dataset, leaving 1,073,522 distribution points. Manual cleanup of incorrectly synonymised species was not performed, as only the genus level was considered.

Intermediate analyses with uncleaned or only partly cleaned data showed that these datasets would have led to different results (Figures S1–S15 illustrate this). The false occurrence data are prevalent to the extent that they blur any meaningful result of the clean data. The usability and consequently the sustainable success of large data repositories such as GBIF will thus in the future largely depend on the effort put into the curation of the data. Currently, these data should only be used with caution (Zizka et al., 2020), and a meaningful dataset can only be extracted via several filtering steps, as seen in this study.

### 3.2 C_4_ eudicot and C_4_ grasses comparison

Species richness is a commonly used measure of biodiversity (Albrecht et al., 2021; Gould, 2000). Richness maps are used to explore patterns of richness and help to investigate the processes that shape those patterns. The species richness maps of C_4_ grasses and C_4_ eudicots show the generally higher species diversity of C_4_ grasses (Figure 2a‐d).

C_4_ eudicots and C_4_ grasses considered together, two regions stood out with a high C_4_ species richness: Mexico/Southern United States and Australia (Figure 2a,c). Additionally, when considering species richness per botanical country, South America can also be identified as a region rich in C_4_ plant species (Figure 2b,d). For the C_4_ eudicots, the hotspot of a high diversity at generic and family rank was in Mexico and extended further north into the United States, where deserts and xeric shrublands prevail, and the Australian hotspot lied in the deserts and xeric shrublands of Central Australia, but extended also into the (sub)tropical region in the north and the Mediterranean region in the west (Figures 2e,f and 4c).

C_4_ grasses showed four diversity hotspots: (1) the tropical and subtropical open coniferous forests, as well as the adjacent deserts of thorn scrubs with fleshy plants and pastures at slightly higher elevations of Mexico, where temperate to semi‐arid climate prevails; (2) the tropical and subtropical grasslands and shrublands of Queensland, Australia; (3) South America; and (4) Africa in tropical and subtropical grasslands, savannas and shrublands (Figure 2a,b). Since India is not divided into provinces like the other tropical countries, it stands out due to its size and the associated high number of species. Looking at the more detailed species richness map with a resolution of 100 × 100 km grid cells, India as a region does not seem to have a high C_4_ species richness (Figure 2a,b). This emphasises the importance of considering different scales and resolutions when analysing ecological patterns.

The rank difference of C_4_ eudicots versus C_4_ grass species occurrence revealed the particular importance of deserts and xeric shrublands (e.g., Africa, Arabian Desert, Asia) and the temperate northern hemisphere (e.g., Mediterranean) for C_4_ eudicots; and the Afrotropical realm (particular Madagascar) and the tropical zone of South America, Southeast Asia and Australia for C_4_ grasses (Figure 2g).

**FIGURE 3:**
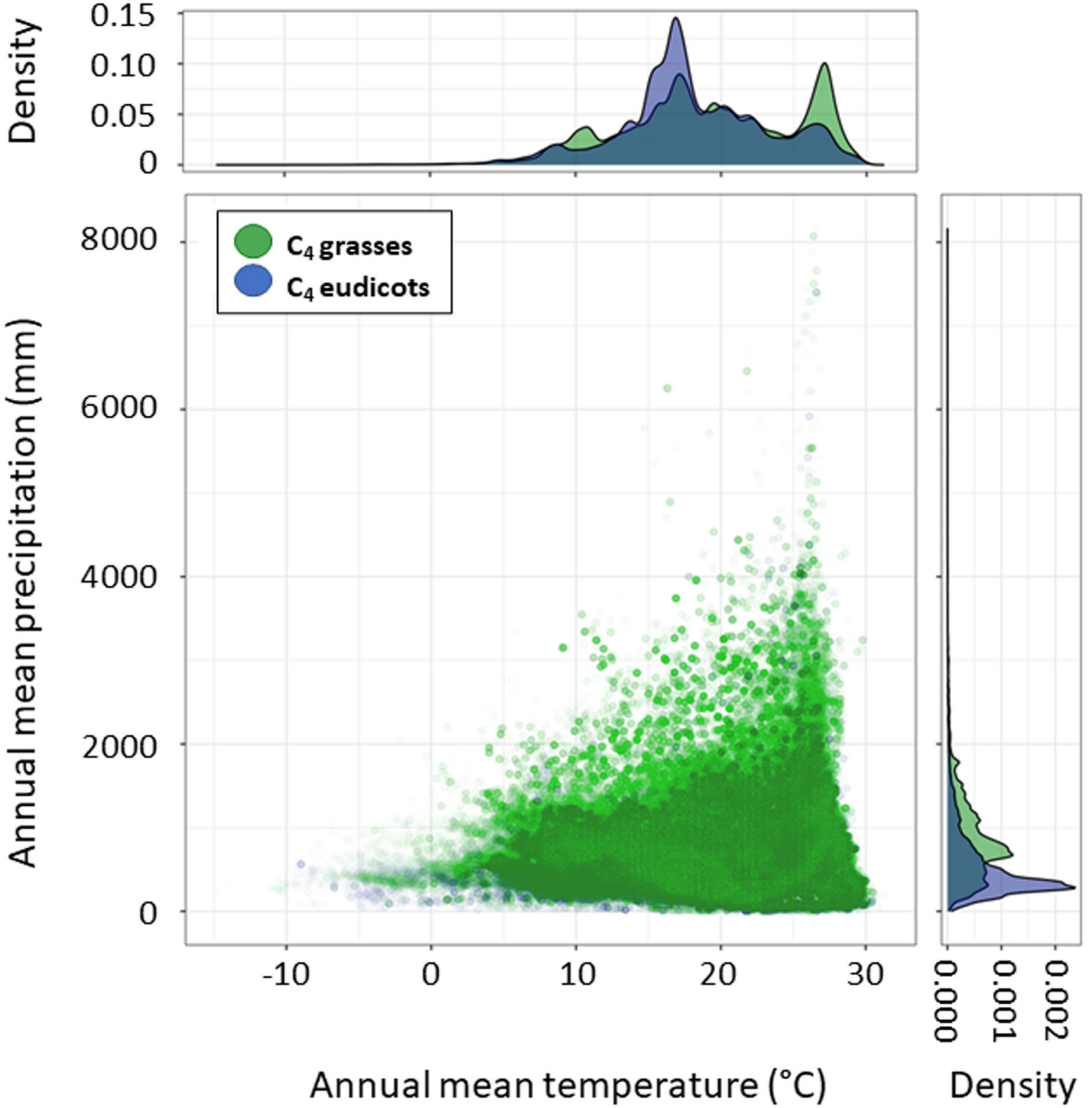
Scatterplot showing annual mean temperature (°C) and annual mean precipitation (mm) data of C_4_ grasses (green) and C_4_ eudicot (blue) occurrence points.

Both, C_4_ grasses as well as C_4_ eudicots, occurred in a wide range of annual mean temperatures from 1 to 31.2°C and 6 to 30.5°C (95% interval), respectively (Figure 3). The median temperature was 19.0°C for C_4_ grasses and 17.4°C for C_4_ eudicots. C_4_ grasses, in addition to increasing in the range of 15–18°C, had a second steep increase in occurrence records ranging between 27 and 30°C, dominated by the subfamilies Chloridoideae and Panicoideae. That last peak could not be observed in the C_4_ eudicots. Occurrence points of C_4_ grasses were found in a broad niche of annual mean precipitation profiles, from 0 to approx. 2000 mm (95% interval). However, the predominant occurrence of C_4_ grasses tended to be in the semi‐wet areas. An increase in occurrence points was seen in the range between 600 and 900 mm, with the median of 772 mm. C_4_ eudicots, on the other hand, occurred in distinctly less precipitation areas, with a median of 394 mm (Figure 3). An increase of C_4_ eudicot occurrence points was observed in regions with approx. 300–600 mm precipitation/year (Figure 3). On a per‐continent basis, the occurrence of C_4_ grasses and C_4_ eudicots differed most prominently in Europe and Africa (Figure S16). In Africa, C4 eudicots show a higher density in areas with <500 mm precipitation, especially in regions with cooler temperatures. In Europe, C_4_ eudicots show a higher density in areas with <400 mm precipitation and warm temperature.

Overall, the diversity and abundance of C_4_ plants increased with increasing annual mean temperature and dry season and decreased with increasing cold temperatures and rainfall. For C_4_ grasses, there was a trend to wetter areas than in C_4_ eudicots. Areas with cool and dry conditions are primarily colonised by C_4_ eudicots.

### 3.3 Diversity of C_4_ eudicots

**FIGURE 4:**
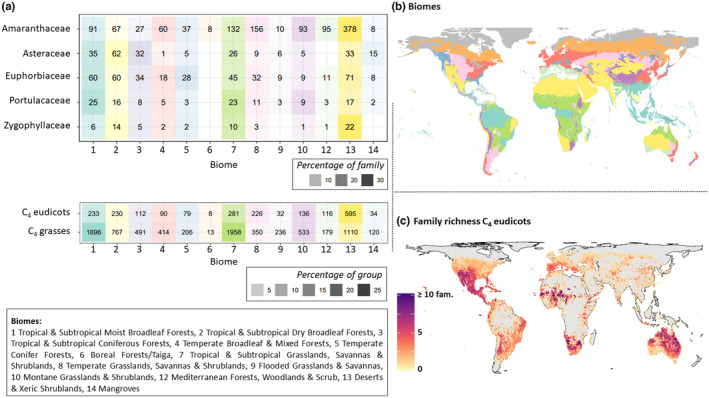
(a) The table shows the number of species of a family within a biome (Terrestrial ecoregions of the world (Olson et al., 2001)). Species are considered present in a biome if at least 5% of the distribution points are in that biome. The five C_4_ eudicot families with the highest number of species in this study are shown (Figure 1a), as well as C_4_ eudicots and C_4_ grasses in general. Colours correspond to the biomes shown in (b). Saturation shows the percentage of species per family or per group (C_4_ eudicots, C_4_ grasses) in each biome. (b) Terrestrial biomes of the world (Olson et al., 2001). (c) Global richness map of C_4_ eudicot families, showing the number of families occurring in each grid (100 × 100 km).

Mapping C_4_ occurrence points at family level revealed many C_4_ eudicots hotspots of high taxonomic diversity at higher ranks with C_4_ species from greater than or equal to seven families occurring in the same area (Figure 4c). These hotspots were Mexico/Southern United States, Australia, South and West Africa, and South America. In South America, the hotspot was located in the montane grasslands and shrublands of Argentinian Patagonia, whereas the Australian hotspot at the family level expanded into the tropical and subtropical grasslands and shrublands.

At the genus richness of C_4_ eudicots, additional diversity hotspots were retrieved: in Asia the temperate grasslands, savannas and shrublands and the Altai‐Sayan mountain range and in Europe in the Mediterranean shrublands (Figure 2e,f).

Amaranthaceae, Asteraceae, Euphorbiaceae, Portulacaceae and Zygophyllaceae are the five eudicot families with the highest numbers of C_4_ species (Figure 4a). While C_4_ Amaranthaceae showed high species richness in many different biomes, the major biomes of C_4_ eudicots were tropical and subtropical moist and dry broadleaf forests (Biome 1 & 2), tropical, subtropical and temperate grasslands, savannas and shrublands (Biome 7 & 8), and deserts and xeric shrublands (Biome 13).

**FIGURE 5:**
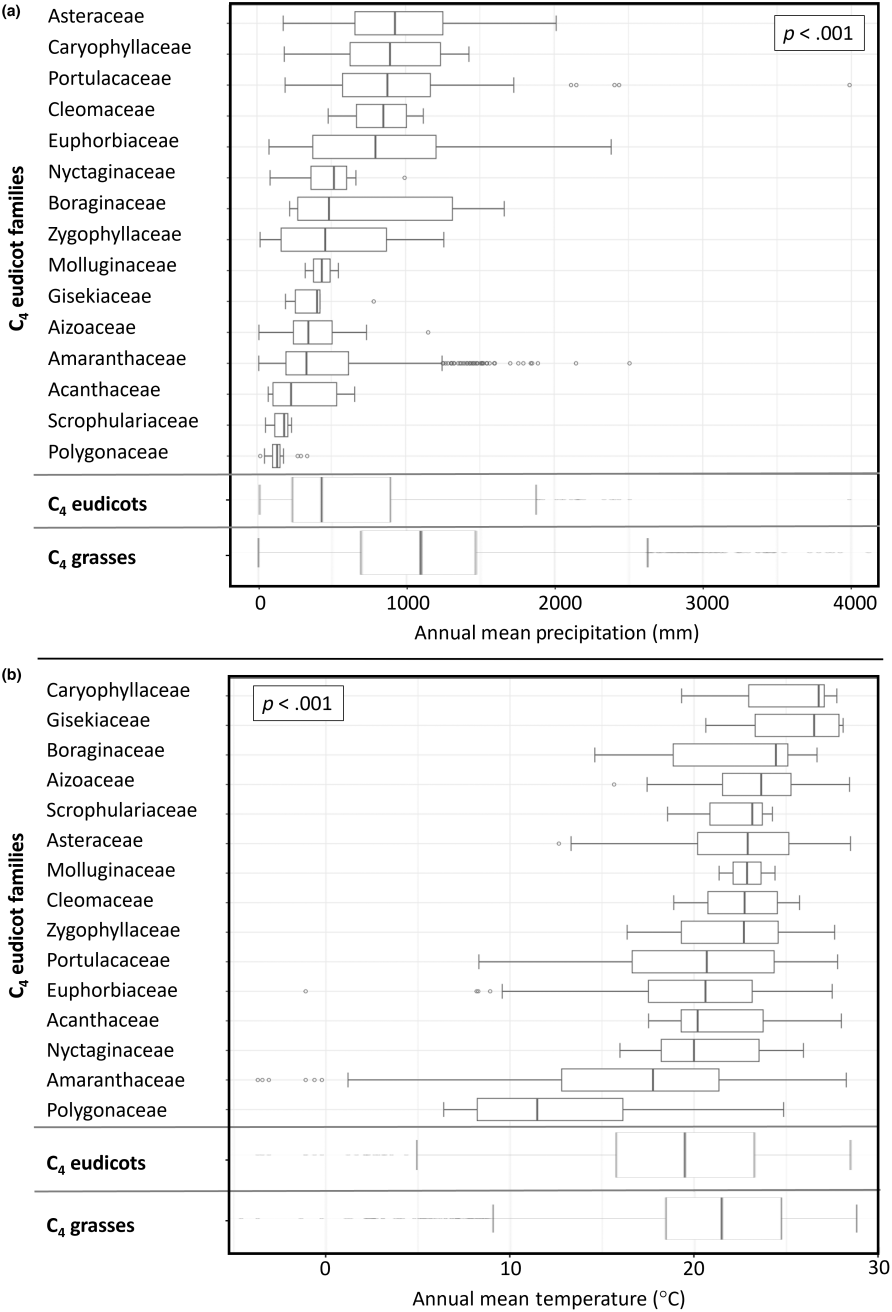
Differences in climatic preferences of the C_4_ species in eudicot families, and C_4_ eudicots and C_4_ grasses in general. A Kruskal–Wallis test was used over C_4_ eudicot families: *p* < .001 statistically significant. (a) Annual mean precipitation (b) and annual mean temperature.

We found statistically significant differences in precipitation and temperature between C_4_ eudicot families (Kruskal–Wallis: for annual mean precipitation – chi‐squared = 231.34, df = 14, *p*‐value <2.2e‐16; for annual mean temperature – chi‐squared = 161.73, df = 14, *p*‐value <2.2e‐16) and between C_4_ eudicots and C_4_ grasses (Mann–Whitney *U* test: for annual mean precipitation – *p*‐value <2.2e‐16; for annual mean temperature – *p*‐value <2.2e‐16). These results point towards a wide adaptation range to diverse environmental conditions among the families and both groups (Figure 5).

Focusing on the five families (Amaranthaceae, Asteraceae, Euphorbiaceae, Portulacaceae and Zygophyllaceae) considered as species‐rich per grid in Figure 4, we noticed that Amaranthaceae differed significantly (Post‐hoc Tukey test, *p*‐value of <.01) in the annual mean temperature and precipitation range from the other four families, except when comparing Amaranthaceae and Zygophyllaceae for the precipitation range (Post‐hoc Tukey test, *p*‐value of 1). Further multiple comparisons between the four other plant families showed no significant difference in terms of temperature (Post‐hoc Tukey test, *p*‐value of >.06). In terms of precipitation range, the comparisons between Zygophyllaceae‐Asteraceae, Zygophyllaceae‐Euphorbiaceae and Zygophyllaceae‐Portulacaceae showed a significant difference (Post‐hoc Tukey test, *p*‐value of <.01). Amaranthaceae had the lowest mean value of 17.77°C of these five families. Furthermore, the mean value of annual precipitation in Amaranthaceae was 333 mm. The mean value of the preferred annual mean temperature for Asteraceae was 22.91°C, and annual precipitation of 929 mm, occurring in wetter and warmer areas compared to the other four families. C_4_ species of Euphorbiaceae preferred rather wet areas (mean = 797 mm) with a wide interquartile range and temperatures that intersect with the preferred areas of Amaranthaceae and Asteraceae (mean = 20.62°C). A similar pattern was retrieved for C_4_ species of the Portulacaceae. Their preferred temperature lied between the values of Amaranthaceae and Asteraceae (mean = 20.69°C) and the C_4_ species of Portulacaceae occurred in areas with an annual precipitation of 878 mm. Zygophyllaceae together with Asteraceae preferred the warmest areas among these five families (Zygophyllaceae: mean = 22.71°C), and the mean value of the preferred annual precipitation for Zygophyllaceae was second lowest at 459 mm.

Families with only one C_4_ lineage (genus) stood out among the others. In Polygonaceae, a single C_4_ genus *Calligonum* that occurs in the cold deserts of Central Asia was particularly conspicuous in climatic preferences with the lowest mean value of 11.48°C and a precipitation preference in the very dry range (mean = 136 mm). A different picture was observed in C_4_ species of the family Caryophyllaceae which also contains only one C_4_ genus, *Polycarpaea*. These C_4_ species preferred comparatively warmer (mean = 26.72°C) and wetter regions (mean = 895 mm).

### 3.4 Traits

A total of 394 (39% of total) succulent C_4_ species were recorded in eight eudicot families (Aizoaceae, Amaranthaceae, Caryophyllaceae, Gisekiaceae, Polygonaceae, Portulacaceae, Molluginaceae, Zygophyllaceae). These are distributed around a mean value of annual precipitation of 444 mm, which is higher than the mean for all C_4_ eudicots. Salt tolerance is documented in seven families with about 485 (49%) species, 333 (33%) of which are succulents from five families (Aizoaceae, Amaranthaceae, Polygonaceae, Portulacaceae, Zygophyllaceae). The biochemical subtypes were also examined. 459 (46%) investigated species within 11 families were retrieved to have the NAD‐malate enzyme as the predominant decarboxylase. In contrast, 559 (56%) species within ten families exhibited the NADP‐ME biochemical subtypes.

**FIGURE 6:**
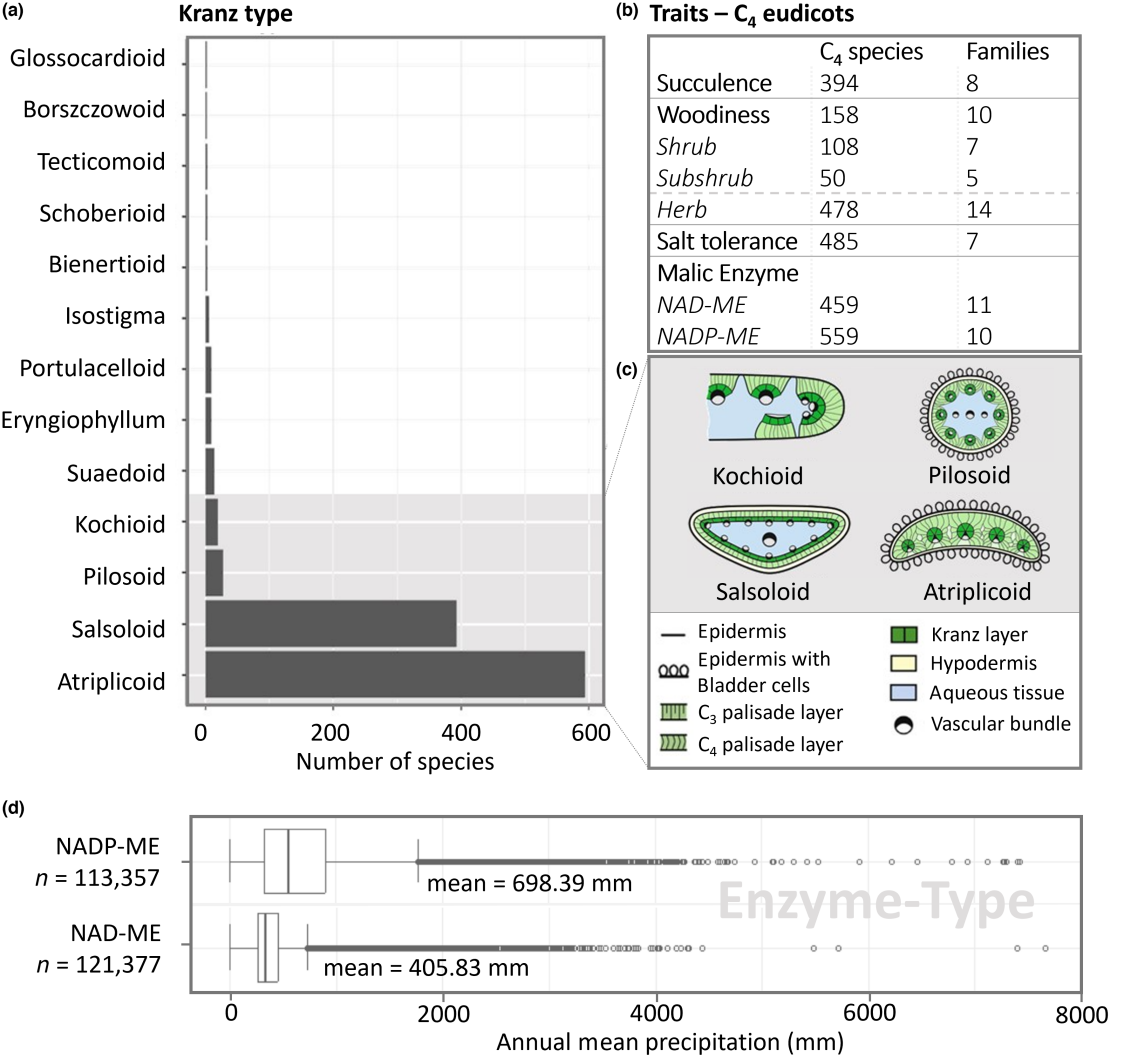
(a) Distribution of Kranz types present in C_4_ eudicots. (b) Distribution of the traits summarised within the C_4_ eudicots. Separated into the number of C_4_ species and the number of families in which the traits occur. (c) Schematic illustration of the four common C_4_ leaf types (Schematic drawings for illustration of leaf anatomical types are adapted from Bohley et al., 2015). (d) Range and mean of annual precipitation of species with NADP‐ME and NAD‐ME as primary decarboxylating enzymes.

Most C_4_ eudicot species showed the classical atriplicoid leaf anatomy without or only little accompanied water storage tissue (Figure 6c). This anatomy with minor differences was also predominant in C_4_ grasses (Edwards & Voznesenskaya, 2011). In cases of succulence, there was often a deviation from atriplicoid anatomy. A high diversity in the succulent leaf anatomy of C_4_ eudicots was observed, with most C_4_ leaf types occurring only in a few species (Figure 6). However, the salsoloid leaf anatomy was clearly the most common leaf type among succulent C_4_ eudicots, not only in the Amaranthaceae but also in Aizoaceae and Polygonaceae (Table S1). Among the succulent species, ca. 3% were stem succulents, while the rest were leaf succulents. The diversity of the leaf anatomy was closely linked to succulence and non‐succulent leaves were usually atriplicoid. Eudicot C_4_ species with NAD‐ME as the primary decarboxylating enzyme are distributed in areas with significantly lower annual precipitation than NADP‐ME subtype eudicot C_4_ species (*F* = 26,936, *p*‐value <2.2e‐16; annual mean precipitation of 405.83 mm versus 698.39 mm).

### 3.5 Results of the literature survey on geographical origin of C_4_ photosynthesis

Seventeen genera out of eight C_4_ eudicot families were distributed in the diversity hotspot of Mexico and Southern United States (Table 2). Five of these genera encompassed more than ten C_4_ species found in the area, with six C_4_ genera originated in the area. Out of these in situ C_4_ genera two, *Euphorbia* and *Pectis*, are species‐rich.

**TABLE 2:** List of eudicots C_4_ genera occurring in the diversity hotspots Mexico/Southern United States and Australia according to our occurrence points and of C_4_ genera with molecular evidence of C_4_ in situ origin within the remaining diversity hotspots. Genera that are species‐rich (>10 species) in the respective region are indicated in bold (**x**). Genera that likely originated in the respective region are marked with *.

Family	Genus	Mexico/south US	Australia	C_4_ origin	References
Acanthaceae	*Blepharis*			Africa	Fisher et al. (2015)
Aizoaceae	*Sesuvium*			Africa	Bohley et al. (2015)
	*Trianthema*	x	x	Africa	Bohley et al. (2015)
	*Zaleya*		x	Africa	Bohley et al. (2015)
Amaranthaceae	*Alternanthera*	x	x	More likely origin: South American tropics	Sage et al. (2007), Sánchez‐del Pino et al. (2012)
	*Amaranthus*	**x**	x	Origin unknown	
	*Atriplex*	**x**	**x**	Continental Asia	Žerdoner Čalasan et al. (2022)
	*Froelichia*	x		Origin unknown	
	*Gomphrena*	x	**x**	Origin unknown	
	*Guilleminea*	x		Origin unknown	
	*Suaeda*	x		Origin unknown	
	*Tecticornia*		x*	Australia	Shepherd et al. (2005), Voznesenskaya et al. (2008)
	*Tidestromia*	x*		Mexico/South US	Sánchez‐del Pino and Motley (2010)
Asteraceae	*Flaveria*	x*		Mexico/South US	Morales‐Briones and Kadereit (2023)
	*Glossocardia*		x	Origin unknown	
	*Pectis*	x*		Mexico/South US	Hansen et al. (2016)
Boraginaceae	*Euploca*	x	x	Origin unknown	
Caryophyllaceae	*Polycarpaea*		x	Origin unknown	
Cleomaceae	*Cleome*		x	Origin unknown	
Euphorbiaceae	*Euphorbia*	x*	x	Mexico/South US (*Euphorbia* subg. *Chamaesyce* sect. *Anisophyllum* subsect. *Hypericifoliae)*	Yang and Berry (2011), Horn et al. (2014)
Gisekiaceae	*Gisekia*			Africa	Bissinger et al. (2014)
Nyctaginaceae	*Allionia*	x*		Mexico/South US	Douglas and Manos (2007)
	*Boerhavia*	x*	x	Mexico/South US	Douglas and Manos (2007)
Portulacaceae	*Portulaca*	**x**	x	Origin unknown	
Scrophulariaceae	*Anticharis*			Africa	Khoshravesh et al. (2012)
Zygophyllaceae	*Kallstroemia*	x		Origin unknown	
	*Tribulopis*		x^(^*^)^	Less clear origin: Australia	Lauterbach et al. (2019)
	*Tribulus*		x	Origin unknown	
	*Zygophyllum*			Africa (*Z. simplex*)	Bellstedt et al. (2012)

Sixteen genera out of ten C_4_ eudicots families were distributed in the diversity hotspot Australia (Table 2). Two of these contained more than ten C_4_ species found in the area, with only one species‐poor C_4_ lineage (C_4_ *Tecticornia*) unequivocally originating in the area. *Atriplex* and *Gomphrena* were the species richest C_4_ genera in Australia. While C_4_ Australian *Atriplex* originated ex situ the area of origin of C_4_ in *Gomphrena* is currently unknown (Table 2 and citation therein). Overall, in 14 cases the area of origin of C_4_ photosynthesis is still insufficiently investigated (Table 2).

## 4 DISCUSSION

We characterised the global occurrence of C_4_ eudicots, identified diversity hotspots and climatic preferences, assigned these to specific functional plant traits and conducted literature research on spatiotemporal histories of C_4_ lineages. The database attached to this paper (Table S1) includes the current knowledge of physiological and morphological traits underlying large‐scale patterns for C_4_ eudicots. Our approach combines the thus far known worldwide distribution of C_4_ eudicots, the evolution of photosynthesis and associated traits and climatic preferences of the individual lineages.

So‐called “big data,” as in our case with many distribution points of many different species from GBIF, are valuable resources for wide‐scale analyses and can provide novel insights. However, accurate and elaborate cleaning of the data is essential to obtain meaningful results (Zizka et al., 2019). Another challenge is that in a project with the scope presented here a verification of all the identifications is unrealistic if not close to impossible. Also, we have to take into consideration the sampling density bias of Europe, North America and Australia over large areas of poorly sampled areas of Africa and Asia and thus have to interpret our findings applying to these regions with caution, which is why we additionally obtained species richness maps with the use of WCVP. While WCVP data are less affected by sampling differences, it comes with its own limitations, such as lower spatial resolution and unevenly sized sample areas (e.g., India sticks out as particular species rich in C_4_ eudicots, particularly due to its size). Despite these challenges, using data from both sources allows for a more comprehensive and cautious analysis, taking into account the strengths and limitations of each dataset. These facts additionally underline the general need of well‐curated data in our biodiversity repositories and collecting efforts to fill the sampling gaps if we want them to be used by a broad community of researchers and to provide useful data for wide‐scale analyses.

### 4.1 Similarities and differences in the global distribution of C_4_ grasses and C_4_ eudicots and their climatic and biome preferences

Both, C_4_ monocots and C_4_ eudicots, include multiple C_4_ lineages. While the C_4_ monocots are distinctly richer in species and all except two belong to the order Poales, the C_4_ eudicots belong to seven angiosperm orders (spanning across basal eudicots, rosids and asterids; Figure 1). Nevertheless, this is not indicative of differences in evolutionary age of C_4_ photosynthesis. In both, C_4_ grasses and C_4_ eudicots, the age of C_4_ origins spans from the Oligocene to the Pleistocene with most origins dating back to the Late Miocene (see Sage, 2017 for a summary), an era of global expansion of C_4_ vegetation due to declining atmospheric CO_2_ combined with global cooling and increase in climate seasonality and aridity (Cerling et al., 1997; Wen et al., 2023).

The global maps of C_4_ species richness (Figure 2a‐c) reveal two shared hotspots for C_4_ grasses and C_4_ eudicots: one in Mexico/Southern United States and one in Australia. In these two regions, C_4_ lineages seem to have diversified more intensively than in other parts of the world, and in case of the C_4_ eudicots, this diversity has been recruited independently from multiple families and even multiple times within one family (Figures 2e,f and 4c; Table 2). Two additional hotspot regions for the C_4_ grasses are found in South and West Africa. However, since Africa is in many areas poorly‐sampled, these two regions might appear as C_4_ grasses hotspots due to being proportionally more densely sampled than other regions in Africa. Additionally, looking at the richness of C_4_ grasses per botanical country, East (Tanzania) and Central Africa also count as C_4_ species‐rich (Figure 2b). The richness maps showing the diversity at the genus (Figure 2e,f) and family level (Figure 4c), however, indicate possible further smaller hotspots for the C_4_ eudicots, in Africa (as in grasses), as well as in Patagonia, Central Asia and the Mediterranean. In these regions, diversification within genera is less prominent, but multiple C_4_ eudicot lineages evidently colonised these areas as well (Figures 2e,f and 4c).

Whether these regions of high diversity of C_4_ lineages represent areas of C_4_ origin or were just preferably colonised by already existing C_4_ lineages or both need to be evaluated for each C_4_ lineage and region in a phylogenetic and biogeographical context (see below).

Generally, both C_4_ grasses and C_4_ eudicots showed broad climatic ranges with a large overlap. On average C_4_ grasses occurred in only slightly warmer but distinctly wetter areas than C_4_ eudicots (Figure 3). The occurrence of C_4_ grasses increases in regions with an annual rainfall of around 800 mm (Figure 3) and warm temperatures coupled with high insolation – conditions common in the southern hemisphere (Still et al., 2014). Where these climatic conditions are met C_4_ grasses not only tend to show a high species diversity but often also dominate the vegetation, especially when fires occur regularly (Hoetzel et al., 2013; Sage, 2004; Still et al., 2014). Here are three examples where this is the case: (i) C_4_ grasslands in the highveld of southern Africa, dominated by *Hyparrhenia hirta* (L.) Stapf (Panicoideae) and *Sporobolus pyramidalis* P.Beauv. (Chloridoideae) receive an annual mean precipitation between 400 and 900 mm mainly during the warm summer months (Bond, 2008; Low & Rebelo, 1996; Mills & Cowling, 2006); (ii) species‐rich C_4_ grasslands at the tropical Sudanian savanna near the Volta‐, Benue‐ and Niger‐River experience a peak of summer precipitation of 600 mm in the north and 1000 mm in the south and the West African monsoons that occur between June and August result in warmer and wetter summers that support C_4_ vegetation (Olusegun et al., 2018); (iii) species‐rich C_4_ grasslands in north‐east Queensland and the Northern Territory, Australia, are associated with the tropical to subtropical climate along the coastal strip, the warm and wet summer months (December–February) and the Australian monsoon bringing up to 1300 mm rainfall (Ondei et al., 2016).

C_4_ eudicots colonise predominantly dry to very dry areas with 80% of the occurrences in areas with <800 mm precipitation. One prominent example of an area where C_4_ eudicots show a higher diversity is the cold deserts of Eurasia, with temperatures below the freezing point for an extensive period of time throughout the year (Johnston, 1996; Rudov et al., 2020; Winter, 1981). These are dominated by woody (sub)shrubs *Haloxylon persicum* and *H. ammodendron* as well as *Calligonum aphyllum*, *C. mongolicum* and *Anabasis brevifolia*. All five C_4_ species represent an integral part of the cold desert vegetation and no closely related C_3_ relatives are known from either of these genera (Kürschner, 2004; Lauterbach et al., 2019; Wu et al., 1994–2013). Less prominent C_4_ floral elements of cold Central Asian deserts include species‐poor genera such as *Horaninovia*, *Iljinia*, *Nanophyton*, *Piptoptera*, *Pyankovia*, *Turania* and *Xylosalsola*. These are together with *Anabasis* and *Haloxylon* all members of an evolutionary old C_4_ lineage within Salsoloideae (Amaranthaceae) that likely spread into the cold desert areas several times independently (Akhani et al., 2007; Kadereit et al., 2012). The biogeographically most comprehensively studied genus among these is *Anabasis*, which revealed the adjacent hot deserts of the Irano‐Turanian Provinces as source areas for the species occurring in the cold deserts of the Mongolian Province (Lauterbach et al., 2019). Another example of vegetation with high diversity of C_4_ eudicots is the hot deserts of Central Australia where certain species of the large genus *Atriplex* such as *A. holocarpa*, *A. lindleyi* and *A. vesicaria* are highly abundant (Wilson, 1984).

In terms of preferred biomes, our analyses revealed that C_4_ grasses are most common in (sub)tropical grasslands, savannas and shrublands, whereas the highest species diversity of C_4_ eudicots was recorded from deserts and xeric shrublands (Figure 4). Both, C_4_ grasses and C_4_ eudicots, are fairly common even in (sub)tropical moist broadleaf forests. All these biomes, except the latter, are demarcated by scarcity and/or seasonality of precipitation. For example, one part of C_4_ grasses occurs in the rainforests of the Australasian realm such as tropical and Central Range montane rainforests of Queensland. Another part occurs in the Indomalayan realm, such as Borneo lowland rainforests, Kayah‐Karen and Sri Lanka montane rainforests and South Taiwan monsoon rainforests. Expectedly, both C_4_ groups are scarcer in water‐rich and cooler biomes probably because the C_4_ syndrome is less advantageous and C_3_ species are more competitive. In regions where C_3_ trees dominate, the C_4_ syndrome might be a disadvantage due to the limitations of the higher ATP‐demand of this pathway in shady habitats (Ehleringer & Björkman, 1977; Sage & McKown, 2006). Within C_4_ eudicots, Amaranthaceae are ecologically the most diverse and are the only C_4_ eudicot clade found at higher latitudes in boreal forests.

### 4.2 Did C_4_ lineages originate in the diversity hotspots or did C_4_ lineages colonise these areas?

#### 4.2.1 C_4_ hotspot Mexico/southern United States

The C_4_ hotspot in Mexico and the Southern United States encompasses mostly deserts and xeric shrublands with different climatic regimes. This southern tip of the Nearctic realm comprises warm deserts such as Mojave Desert, Sonoran Desert, Chihuahuan Desert as well as cold deserts of the Great Basin, and large adjacent and equally diverse semi‐desert areas (Laity, 2008). This climate and habitat diversity probably promoted speciation, making these areas particularly species‐rich. About two‐thirds of the flora is endemic to this region and the most common plant families represented in this flora are Cactaceae, Asteraceae and Boraginaceae (Villarreal‐Quintanilla et al., 2017). The majority of endemic species and also the most widely distributed species that typify these landscapes such as *Ambrosia monogyra*, *Artemisia filifolia* and *Flourensia cernua* (Asteraceae), *Ephedra torreyana* (Ephedraceae), *Larrea tridentata* (Zygophyllaceae), *Penstemon thurberi* (Plantaginaceae), *Poliomintha incana* (Lamiaceae), *Prosopis glandulosa* and *Psorothamnus scoparius* (Fabaceae) and *Yucca elata* (Asparagaceae, Shreve, 1939), however, do not perform C_4_ photosynthesis. Nevertheless, here we recorded many C_4_ species from 17 eudicot genera belonging to eight different families (Table 2), supporting the findings of Sage et al. (2011) that Mexico and the Southern United States are a hotspot of C_4_ lineage diversity. For at least six of these lineages, the current molecular phylogenies deliver sufficient evidence for an in situ origin of C_4_ photosynthesis within this area, with two of these being species‐rich (Table 2).

One is the neotropical genus *Pectis* (Tageteae, Asteraceae) which includes approximately 90 C_4_ species and is represented by about 47 species in Mexico and the Southern United States (Hansen et al., 2016). The sister genus *Porophyllum* performs C_3_ photosynthesis and is also distributed in tropical and subtropical America. Hansen et al. (2016) show that the transition to C_4_ photosynthesis occurred most likely during the Late Miocene in the stem lineage of *Pectis* which was probably distributed in North/Central Mexico. Within the mega‐diverse family Euphorbiaceae the evolution of carbon concentrating mechanisms led to diversification bursts (Horn et al., 2014). Here we find the second example of in situ origin of C_4_ photosynthesis in Mexico/Southern United States with subsequent diversification (Horn et al., 2014; Yang & Berry, 2011). The C_4_ pathway evolved only once within the subgenus *Chamaesyce* at the stem of section *Anisophyllum* subsection *Hypericifoliae* during the Mid‐Miocene and gave rise to approximately 350 C_4_ species, which constitutes the largest eudicot C_4_ lineage known thus far. For the well‐known C_4_ model *Flaveria* (Asteraceae; Monson & Moore, 1989; Sage et al., 2014) the phylogenetic tree topology suggests that the C_4_ pathway originated in this area, likely during the Pliocene (Morales‐Briones & Kadereit, 2023). The genus comprises 21 species, mainly distributed in Southern North America, with few species occurring in the Caribbean and South America (Powell, 1978). The C_4_ genera of Nyctaginaceae, *Boerhavia* and *Allionia*, belong to the “North American xerophytic clade” of the family (Douglas & Manos, 2007; Khoshravesh et al., 2020). This clade likely diversified in the deserts of the southwestern United States and northwestern Mexico because all genera are either confined to or represented in the area (Douglas & Manos, 2007). *Boerhavia* subsequently spread and diversified in subtropical regions worldwide. The situation in the species‐rich *Euploca* (Boraginaceae) is challenging to assess as the published molecular phylogeny lacks support along the backbone (Frohlich et al., 2022). Nevertheless, an in situ origin of the North American C_4_ species seems likely.

Amaranthaceae s.l. are well‐represented in Mexico and the Southern United States with eight genera containing native C_4_ species (Table 2), however, only for *Tidestromia* which consists entirely of C_4_ species that are all but one endemic to the region, an in situ origin seems likely (Sánchez‐del Pino & Motley, 2010). *Froelichia*, *Guilleminea* and *Gomphrena* belong to a species‐rich and widespread C_4_ clade that probably started to diversify during the Mid‐Miocene (Limarino & Borsch, 2020). However, due to insufficient sampling, it is currently impossible to infer whether the C_4_ pathway originated in tropical South America or in subtropical southern North America. Insufficient phylogenetic information also prevents us from inferring the origin of North American *Amaranthus* species (Waselkov et al., 2018). However, since the entire genus exhibits C_4_ and probably originated in South America, *Amaranthus* seems to be a migratory C_4_ lineage in Mexico and the Southern United States. Other migratory C_4_ lineages are *Trianthema* which spread into the area from Africa (Bohley et al., 2015), *Atriplex* which arrived from South America (Žerdoner Čalasan et al., 2022), *Portulaca* (Ocampo & Columbus, 2012; Tamboli et al., 2022) and *Suaeda* (Schütze et al., 2003). The biogeography of the C_4_ genus *Kallstroemia* which is distributed from Central and Southern North America to tropical and subtropical South America remains unclear due to limited phylogenetic support (Lauterbach et al., 2019).

Nowadays, the C_4_ hotspot in Mexico and the Southern United States receives a limited amount of rain, ranging from around 50–250 mm per year (Pearcy & Ehleringer, 1984), due to rain shadow casted by the mountain ranges of Sierra Madre Occidental and Sierra Nevada, and Sierra Madre Oriental on either side, which are of Late Mesozoic and Early Cenozoic age (Dickinson, 2004). The earliest evidence of desertification in this area dates to the Middle Miocene and corresponds with the diversification events of arid‐adapted lineages (Eronen et al., 2012; Hyland et al., 2019; Said Gutiérrez‐Ortega et al., 2018; Vásquez‐Cruz & Sosa, 2020). This refers also to some of the in situ originated C_4_ lineages, such as *Pectis*, *Flaveria* (both Asteraceae) and *Allionia* and *Boerhavia* (both Nyctaginaceae) which have originated and spread since the Mid to Late Miocene (Table 2). We suggest that the overall high diversity of ancestral C_3_ lineages in the area adapted to arid conditions in addition to the high selective pressure in favour of the evolution of a carbon concentration mechanism is responsible for the exceptionally high diversity of C_4_ lineages that originated in Mexico and the Southern United States. In addition to these in situ C_4_ lineages a high number of migratory C_4_ lineages occur finding suitable growing conditions in the area.

#### 4.2.2 C_4_ hotspot Australia

Within Australia, two regions of high C_4_ plant diversity with different precipitation profiles are observed. The first one constitutes deserts and xeric shrublands of the Eremaean floristic region (sensu Ebach et al., 2015), rich in C_4_ *Atriplex* (about 60 species) but also in C_3_ Camphorosmeae (about 150 species) and *Chenopodium* (about 50 species; Kadereit et al., 2005). Most other Australian C_4_ eudicots are restricted to the northern parts of the continent where tropical and subtropical grasslands, savannas and shrublands prevail. Here the biggest C_4_ genera are *Gomphrena* (Amaranthaceae) with 30 C_4_ species, followed by *Euphorbia* (Euphorbiaceae) with seven and *Portulaca* (Portulacaceae) and *Polycarpaea* (Caryophyllaceae) with five C_4_ species each.

While there are many different C_4_ plant lineages known from these areas, the majority of them did not evolve in situ (Table 2), albeit little direct evidence is available, due to the lack of robust phylogenies. Some migratory C_4_ lineages diversified in Australia. C_4_ *Atriplex* lineages, for example, reached Australia at least two times independently – once from the Mediterranean/Pontic region at the end of the Miocene and once from Central Asia at the end of the Pliocene, with the latter, younger one, going through an extensive diversification after reaching the continent (Žerdoner Čalasan et al., 2022). A similar pattern can be inferred from Australian C_4_ Aizoaceae (*Trianthema* and *Zaleya*) with Africa as their source area (Bohley et al., 2015). The spatial and temporal aspects of other C_4_ representatives of Australian flora (*Euploca, Glossocardia*, *Gomphrena*, *Polycarpaea*, *Portulaca* and *Tribulus*) remain unclear. Limited data, however, point towards ex situ evolution of the C_4_ syndrome in these genera.

The only clear C_4_ in situ origin is currently known from *Tecticornia* (Shepherd et al., 2005; Voznesenskaya et al., 2008). This taxon is adapted to hyper‐saline conditions and builds extensive vegetation stands along the edges of Australian inland salt lakes (Shepherd et al., 2004). This genus comprises about 60 species, out of which only a clade of five taxa is known to perform C_4_ photosynthesis. While many C_3_ species have rather restricted distribution areas (which may or may not be a result of lack of surveys in poorly accessible Australian outback), one of the two C_4_ species, *Tecticornia indica*, exhibits a wide distribution range along saline lake shores across the whole continent (Wilson, 1984). Another, albeit less clear example is the small Australian genus *Tribulopis*. Conflicting phylogenetic signals between the nuclear and chloroplast‐encoded genes point towards a complex evolutionary history of this taxon (Lauterbach et al., 2019). Contrarily to *Tecticornia*, here the C_3_ representatives show a wider distribution range, whereas the C_4_ species tend to be geographically restricted (Wilson, 1984). While reasons for this peculiar distribution remain unknown, this example clearly indicates that the factors promoting the evolution of C_4_ photosynthesis are multifold and that each individual C_4_ lineage has its own unique evolutionary history. Both taxa arrived to Australia post‐Miocene (Piirainen et al., 2017; Wu et al., 2018), which coincides with several geological and climate features that initiated and promoted aridification of this continent. These include the northward drift towards the equator, expansion of the Antarctic ice cap, and the formation of the circum‐Antarctic Ocean current and subtropical high‐pressure system (Fujioka & Chappell, 2010; Kemp, 1978).

#### 4.2.3 Other C_4_ eudicot hotspots

The scarce dataset revealed three regions in Africa that seem to favour C_4_ eudicots. The first includes the tropical and subtropical grasslands, savannas and shrublands of Africa. The second C_4_‐rich region is located in south‐east Africa. Here we should mention that the Drakensberg Mountain Centre located in this region is a known biodiversity hotspot, which may or may not influence the number of C_4_ species (Carbutt, 2019; Popp & Kalwij, 2021). The third region is in southwestern Africa (Fisher et al., 2015; Schulze & Schulze, 1976; Vogel & Seely, 1977).

Most C_4_ eudicot species in these regions belong to Acanthaceae (*Blepharis* sect. *Acanthodium*), Aizoaceae, Gisekiaceae (genus *Gisekia*), Scrophulariaceae (genus *Anticharis*) and Zygophyllaceae (Table 2). The estimated centre of in situ origin for C_4_ photosynthesis, with molecular phylogenetic evidence, in *Blepharis* (Fisher et al., 2015), *Anticharis* (Khoshravesh et al., 2012), *Gisekia* (Bissinger et al., 2014), Sesuvioideae (*Sesuvium/Trianthema/Zaleya*; Bohley et al., 2015) and the species *Zygophyllum simplex* (Zygophyllaceae; Bellstedt et al., 2012) appears to be the so‐called “Horn of Africa,” which defines the south of the Sahara‐Sindian region, and the arid southwestern Africa (Bellstedt et al., 2012 and references therein).

In South America, two centres of C_4_ eudicot biodiversity are recognised, both under strong influence of arid desert or steppe climate with a pronounced dry period. These two centres of C_4_ diversity are most evident in *Euploca* (Boraginaceae) and *Portulaca* (Portulacaceae). However, they are also found in other eudicotyledons such as Amaranthaceae, Asteraceae and Euphorbiaceae. *Portulaca* has the highest C_4_ species diversity in north‐eastern Brazil. C_4_ *Portulaca* clade started to radiate in the late Miocene/Pliocene (Ocampo & Columbus, 2012), following the expansion of C_4_ vegetation due to decreased CO_2_ levels and increased aridity (Salzmann et al., 2008; Strömberg, 2011). Nevertheless, the north‐eastern portion of Brazil became consistently arid very recently in geological history – at the end of Younger Dryas (Auler et al., 2004). This leads us to believe that the high C_4_ diversity of *Portulaca* in that region is of refugial origin, as continuous wetter interglacial cycles prior to that diminished any advantage of C_4_ species over their C_3_ congeners. Poorly resolved phylogeny and lack of time divergence estimation preclude us from discussing potential stages in the evolutionary history of *Euploca* (Frohlich et al., 2022). For C_4_ *Atriplex* lineages molecular phylogenetic studies show two long‐distance dispersal events to reach South America – one possibly from continental Asia and one from North America (Žerdoner Čalasan et al., 2022).

It is important to mention that there are also regions, such as the Central Asian Deserts, that are not a main area of origin for C_4_ lineages, but have a high C_4_ lineage diversity due to a lot of migration. However, while the knowledge on the geological history of this region increased dramatically in recent years (Barbolini et al., 2020; Hurka et al., 2019), the evolutionary history of its flora remains largely unknown (Seidl et al., 2021; Žerdoner Čalasan et al., 2021).

### 4.3 Functional traits lead to ecological diversity in C_4_ eudicots

Precipitation and temperature preference among the C_4_ eudicot families differ significantly (Figure 5). This supports the well‐known fact that C_4_ is advantageous under different environmental conditions. Various phylogenetic analyses (cited in Table 2) indicate that different evolutionary context dependencies together with the respective ancestral anatomical and physiological phenotypes are likely to have influenced the evolution of C_4_ and with it associated traits of the plant group. Whether C_4_ evolution improved fitness and evolutionary persistence of a lineage or as a key factor enhanced diversification by opening up new niches is lineage‐specific and depends on C_4_‐associated and other lineage‐specific traits. Universal C_4_‐associated traits, such as Kranz anatomy, therefore represent one of several notable examples of phenotypic convergence across a wide range of functional, biochemical and phylogenetic diversity. While it remains poorly understood how Kranz anatomy was initiated and how it arose in C_4_ plants (Schlüter & Weber, 2020), it required a variety of complex developmental changes and thus possibly represent a bottleneck to the C_4_ origin (Lauterbach et al., 2019). Hence, the high number of independent C_4_ origins and their morphological, ecological and physiological diversity in eudicot lineages is unexpected.

Kranz anatomy is an important unifying trait for almost all C_4_ species, and the majority of species show a similar (so‐called atriplicoid) C_4_ anatomy with Kranz cells (or bundle sheath cells) surrounding the vascular bundle and an outer ring of specialised mesophyll cells (Figure 6c). However, many eudicot C_4_ lineages deviate from this common anatomical type and show additional anatomical specialisations related mostly to leaf or stem succulence. All lineages that combine succulence and C_4_ photosynthesis seem to be derived from ancestrally succulent C_3_ lineages (e.g., *Tetraena simplex* in Zygophyllaceae (Lauterbach et al., 2016), C_4_ *Sesuvium* in Aizoaceae (Bohley et al., 2015), C_4_ *Tecticornia* in Amaranthaceae (Shepherd et al., 2005), and C_4_ lineages of Salsoleae, Camphorosmeae and Suaedeae (Kadereit et al., 2012). The annual mean precipitation for occurrence points of C_4_ eudicot succulents is 444.47 mm which does not indicate that the C_4_ succulents are confined to particularly dry areas. Non‐succulent C_4_ eudicots such as Scrophulariaceae and Acanthaceae can be found in comparably dry (but not in saline) habitats. Sometimes succulence is lost within a C_4_ clade as found in *Bassia* (Kadereit & Freitag, 2011). In the salt‐tolerant C_4_ grass *Spinifex littoreus*, however, C_4_ evolution preceded that of succulence and salt tolerance (Ho et al., 2019; Morrone et al., 2012).

Together with salt tolerance known from most of the succulent C_4_ eudicots (Santos et al., 2016), this combination of traits provides solutions to a broad variety of unfavourable climatic and edaphic conditions, which may explain the ecological diversity of C_4_ eudicots. In some succulent C_4_ eudicots, the C_4_ pathway is combined with weak CAM which might provide a rescue mechanism under severe drought. The best‐studied example is *Portulaca oleracea* (Portulacaceae; Moreno‐Villena et al., 2022), but a co‐occurrence of CAM and C_4_ was also found in species of Sesuvioideae in Aizoaceae (Winter et al., 2021: *Trianthema portulacastrum*; Siadjeu and Kadereit, under review.: *Sesuvium sesuvioides*) and might be more common than initially thought. As these studies require living specimens and a multiplexed experimental design to detect the potential co‐occurrence of CAM and C_4_ the low number of reported cases might be due to lack of data.

According to our results, C_4_ eudicots with NADP‐ME tend to be more prevalent in areas with significantly higher rainfall (mean value = 698.39 mm) than those utilising the NAD‐ME as the decarboxylating enzyme (mean value = 405.83 mm; Figure 6d). This has been thus far supported by studies that comparatively investigated the physiology of NADP‐ME and NAD‐ME C_4_ grasses. While species with the NAD‐ME subtype seem to perform better under drier conditions (Carmo‐Silva et al., 2009; Ghannoum et al., 2002), NADP‐ME species tend to have a more effective CO_2_‐capturing system by keeping stomata open longer under wetter conditions and can more efficiently utilise nitrogen (Pinto et al., 2016; Schulze et al., 1996; Taub, 2000). Within C_4_ eudicots the NAD‐ME subtype seems more common than in C_4_ grasses where the NADP‐ME pathway prevails (Sage et al., 2011). This might also explain why C_4_ eudicots managed to successfully occupy even the most arid regions around the globe, whereas C_4_ grasses are on average found in regions with more rainfall (Figure 5a).

### 4.4 A brief summarising family perspective of C_4_ evolution in eudicots

The morphological, physiological and ecological diversity within C_4_ eudicots is immense. The most diverse plant family is Amaranthaceae, in which C_4_ photosynthesis developed several times independently in different environmental conditions (Kadereit et al., 2003, 2012; Sage et al., 2007). While in Salicornioideae (incl. Salsoloideae, Camphorosmoideae and Suaedoideae) and Chenopodioideae, C_4_ photosynthesis arose in ancestrally subtropical and temperate arid, and predominantly succulent and salt‐tolerant lineages, C_4_ clades within Amaranthoideae more likely originated from tropical ancestors. Both NAD‐ME as well as NADP‐ME pathways are present in multiple lineages of this family and its C_4_ representatives can be found in most biomes, including the C_4_ less‐favourable cool boreal zones. A number of rare features in the context of C_4_ photosynthesis are found only in Amaranthaceae. For example, the occurrence of single cell C_4_ in terrestrial angiosperms occurs only in the genus *Bienertia* and in *Suaeda aralocaspica* (both taxa belong to the tribe Suaedeae; Voznesenskaya et al., 2002). Furthermore, the shift from C_3_ photosynthesis in cotyledons to C_4_ in adult leaves has been so far only reported from various species of Salsoleae (Lauterbach et al., 2017). Two species of *Tecticornia* represent the only known stem‐succulent C_4_ species with window cells in their mesophyll (Marchesini et al., 2014; Moir‐Barnetson, 2014), and the *Salsola divaricata* agg. represents the first C_4_ lineages that was shown to have arisen from ancestral hybridisation events between a C_4_ and a C_3_ lineage within Salsoleae (Morales‐Briones & Kadereit, 2023).

Amaranthaceae are in terms of the C_4_ species rich followed by Euphorbiaceae and Asteraceae (Figure 1). In the former predominantly tropical plant family, the C_4_ syndrome evolved only once resulting in a clade of about 150 species found for the most part, but not exclusively, in seasonally dry and arid zones. In Hawaii, *Euphorbia* species occur in habitats ranging from arid coastal beaches to rainforests. One example is *Euphorbia clusiifolia* Hook. & Arn. (formerly *E. forbesii* Sherff, not accepted by Govaerts et al., 2000), a tree species that grows up to 13 m high and is endemic to the cool, mesic, subtropical forests in the geographically young Hawaiian Islands (Pearcy & Ehleringer, 1984). Further C_4_ trees include *Euphorbia olowaluana* and *E. remyi*, which occur in dry open and subalpine, and humid forests, respectively (Young et al., 2020). These examples strongly indicate that the C_4_ pathway is not limited to herbaceous or shrubby life forms but may also occur in trees, albeit these examples evolved woodiness secondarily from herbaceous ancestors on the island (Zizka et al., 2022). This link is particularly interesting since the secondary evolution of woodiness may in turn be linked to drought adaptation in other lineages (Dória et al., 2018; Hooft van Huysduynen et al., 2021). Many other C_4_ *Euphorbia* species occur in the understory rainforests of Hawaii, where the precipitation is rather high with an average range of 1200 mm to 1800 mm annually (Pearcy & Ehleringer, 1984) – conditions, in which C_4_ photosynthesis does not seem to have a physiological advantage.

In Asteraceae, the C_4_ syndrome originated several times resulting in about 100 species that often sympatrically occur with the C_3_ congeners in dry and arid habitats. Due to the presence of many C_3_–C_4_ intermediate species, *Flaveria* became a model of C_4_ evolution (Monson & Moore, 1989; Sage et al., 2014). The species of the genus *Isostigma*, native to Bolivia, Brazil and North Argentina, show Kranz anatomy in the stems and two different types of Kranz anatomy in leaves (Peter, 2009). The *Eryngiophyllum* type, common in hot and arid conditions, has one Kranz unit per leaf and sclerenchyma tissue, whereas the *Isostigma* type, more common in places with higher precipitation, shows more than one Kranz unit per leaf and no sclerenchyma tissue (Peter & Katinas, 2003). C_4_ representatives of Euphorbiaceae and Asteraceae rely solely on the NADP‐ME pathway with the exception of *Euphorbia mongolica*, which can be found in highly seasonal temperate East Asia (Zang et al., 2021) and exhibits the NAD‐ME pathway.

Preliminary data suggest that C_4_ photosynthesis evolved only once in Caryophyllaceae in the genus *Polycarpaea* somewhen in the Pliocene (Kool, 2012). Apart from one widely distributed C_4_ species, all the other C_4_ species are restricted predominantly to either subtropical forests and (semi)arid zones of Australia or to the western Africa and adjacent regions. The highest species diversity of the C_3_ representatives is the more mesic Canary Islands. Low number of extremely xeric species might be explained by the presence of the NADP‐ME mechanism, which seems less efficient under extremely arid conditions, at least in grasses (Rao & Dixon, 2016). In Boraginaceae, the C_4_ photosynthesis arose probably at least twice independently and while genera *Euploca* and *Heliotropium* are distributed worldwide, the C_4_ representatives of either genus are found predominantly in the seasonally dry and (semi)arid habitats around the globe. A similar pattern is observed also in C_4_ representatives of Cleomaceae, Molluginaceae and Nyctaginaceae, in all three of which the diversity of C_4_ species is markedly lower than that of C_3_ species.

While the majority of Aizoaceae rely on the CAM carbon fixation pathway, there are about 30 species that perform C_4_ photosynthesis. The details of anatomy and biochemistry vary greatly among closely related species with several origins of both NAD‐ME and NADP‐ME pathways (Bohley et al., 2015). The most widely distributed C_4_ species in this group under current taxonomic treatments is *Trianthema portulacastrum*, which can carry out CAM and C_4_ (Winter et al., 2021). While this physiological plasticity possibly facilitated its wide distribution, other widely distributed non‐C_4_ (but also weak CAM; Winter et al., 2019) species such as *Sesuvium portulacastrum* show that C_4_, albeit beneficial, cannot solely explain species' wide distribution. In monogeneric Portulacaceae about two‐thirds of currently known species from the genus *Portulaca* exhibit C_4_ anatomical features using NAD‐ME and NADP‐ME carbon fixation pathways in closely related lineages (Ocampo et al., 2013). Furthermore, many species also show reversible physiological signs of an effective CAM carbon fixation pathway under extreme drought stress (Holtum et al., 2017; Moreno‐Villena et al., 2022). While this physiological plasticity may again explain the wide distribution range of *Portulaca oleracea* complex, its taxonomic uncertainty hampers our understanding of its evolutionary history and its true distribution extent (Ferrari et al., 2020).

While majority of the C_4_ eudicots occur in regions with a moderate drought period, Polygonaceae, Scrophulariaceae and Zygophyllaceae have the highest C_4_ species diversity in much drier arid and semi‐arid regions. The shrubby species of *Calligonum* (Polygonaceae) possess only the NAD‐ME mechanism for carbon fixation (more effective in drier regions), have a salsoloid Kranz type and are found only in cold and dry deserts of Eurasia and Africa (Sage et al., 2011; Sage, 2017; Pyankov et al., 2000, 2010; Muhaidat et al., 2012; Figure S12b; Table S1). In Africa, it shares its distribution range with the only C_4_ taxon from Scrophulariaceae, *Anticharis*, whose four species are restricted to this warm region and exhibit NAD‐ME mechanism for carbon fixation as well. In Zygophyllaceae, the C_4_ photosynthesis developed several times independently and majority of the representatives are found in hot deserts around the world (Lauterbach et al., 2019). Interestingly, apart from one species (*Tetraena* (=*Zygophyllum*) *simplex*) all C_4_ species of Tribuloideae seem to possess the NADP‐ME subtype. While it remains unclear why this is the case, *Tetraena simplex* is also the only species whose C_4_ anatomy resembles the kochioid Kranz type, while the rest of the taxa show atriplicoid Kranz type. This is not the case in other taxa, where NADP‐ME and NAD‐ME are not restricted to a particular Kranz anatomy.

C_4_ representatives of Acanthaceae and Gisekiaceae have a comparable distribution area and are found across seasonally dry to arid habitats of (sub)tropical Africa and southwestern Asia. In both families the C_4_ syndrome arose probably only once (Bissinger et al., 2014; Fisher et al., 2015). While *Blepharis* (Acanthaceae) includes C_3_ representatives as well, and both NAD‐ME and NADP‐ME pathways are known from its C_4_ representatives, in *Gisekia* (monogeneric Gisekiaceae) all currently known species perform NAD‐ME‐based C_4_ photosynthesis. Despite this restriction, both taxa are found along a wide range of open and often disturbed arid to mesic habitats (Bissinger et al., 2014).

## 5 CONCLUSION/OUTLOOK

Although the climatic ranges of C_4_ grasses and C_4_ eudicots mostly overlap, our findings suggest that C_4_ eudicots tend to inhabit substantially drier regions than their C_4_ monocot counterparts. This could be due to the numerous phylogenetically less constrained morphological, ecological and physiological adaptations to harsh environments present in C_4_ eudicots. C_4_ eudicot lineages employing the NAD‐ME decarboxylating enzyme inhabit notably drier regions in comparison to those utilising the NADP‐ME decarboxylating enzyme. We conclude that C_4_ evolution in most eudicot lineages occurred in ancestral drought‐adapted clades, thereby facilitating the expansion of such plants in these habitats and allowing them to colonise even drier areas. We identified primary hotspots of C_4_ eudicots that are corroborated by phylogenetic studies as source and sink areas of C_4_ diversity, respectively: the arid regions in Mexico/Southern United States, Australia and Central Asia, where multiple C_4_ eudicot lineages diversified independently possibly due to increasingly drier environmental conditions. Our literature review on evolutionary histories of individual taxa in these regions, albeit scarce, indicates that evolutionary history of C_4_ elements in these regions differs greatly. Mexico and the Southern United States exhibit a high number of C_4_ lineages that originated in situ, whereas high C_4_ diversity in Australia and Central Asian deserts results primarily from the secondary migration of C_4_ lineages into these areas.

## AUTHOR CONTRIBUTIONS

**Jessica A. Berasategui:** Conceptualization (equal); data curation (lead); formal analysis (lead); visualization (lead); writing – original draft (lead); writing – review and editing (lead). **Anže Žerdoner Čalasan:** Writing – original draft (supporting); writing – review and editing (supporting). **Alexander Zizka:** Conceptualization (supporting); formal analysis (supporting); visualization (equal); writing – review and editing (supporting). **Gudrun Kadereit:** Conceptualization (equal); formal analysis (supporting); writing – original draft (supporting); writing – review and editing (supporting).

## CONFLICT OF INTEREST STATEMENT

The authors declare no conflict of interest.

## Supporting information


Appendix S1

## Data Availability

The data that support the findings of this study are available in the supplementary material of this article.
