# Supplementary material for: Global distribution, climatic preferences and photosynthesis‐related traits of C4 eudicots and how they differ from those of C4 grasses
Source: Ecol Evol. 2023 Nov 12;13(11):e10720. doi: 10.1002/ece3.10720 (PMC10641307; doi:10.1002/ece3.10720)

Figures A1-15: Species richness maps showing the impact of data cleaning of all 15 families. (a) Map showing the uncleaned distribution points of the families. (b) Distribution points of the families after data cleaning.

Figure A1

Acanthaceae

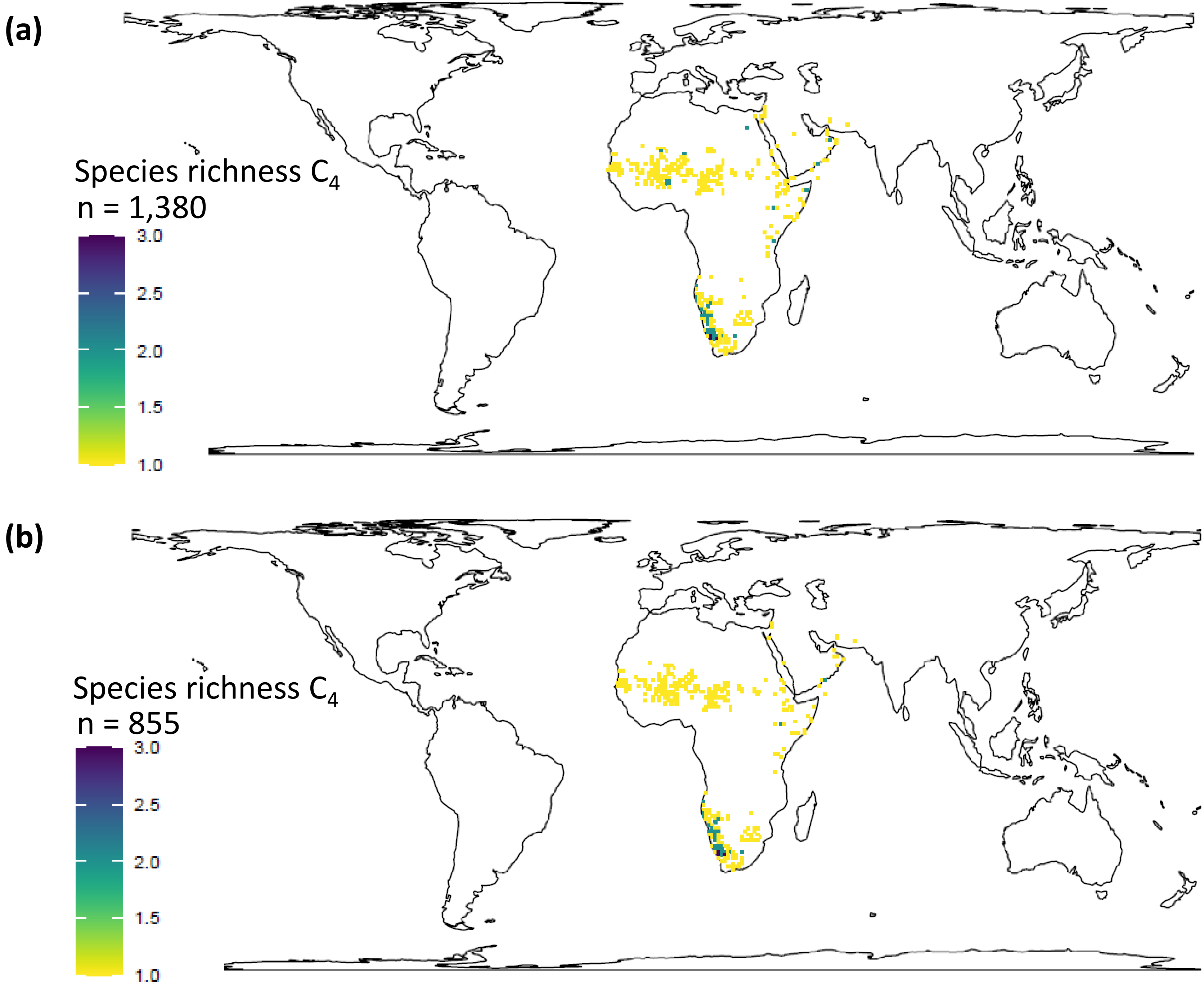

Figure A2

Aizoaceae

(a)

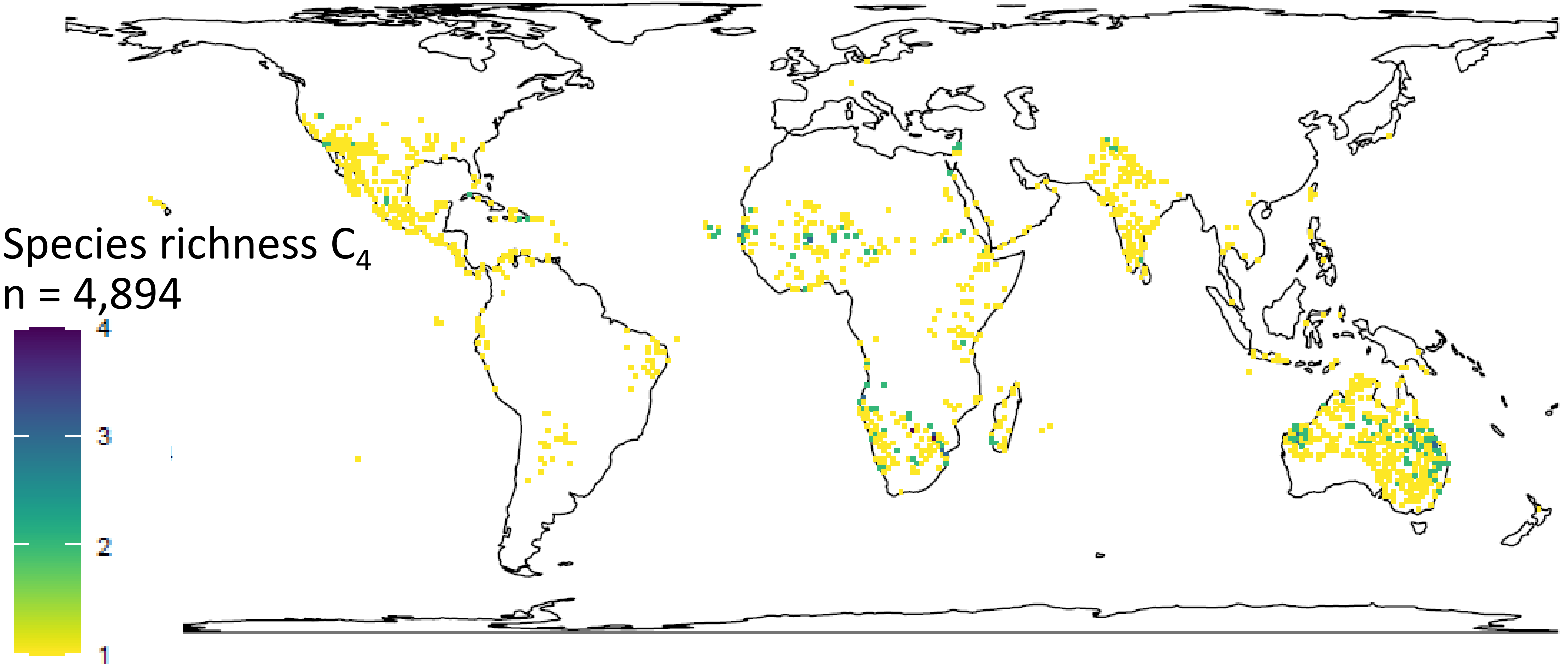

(b)

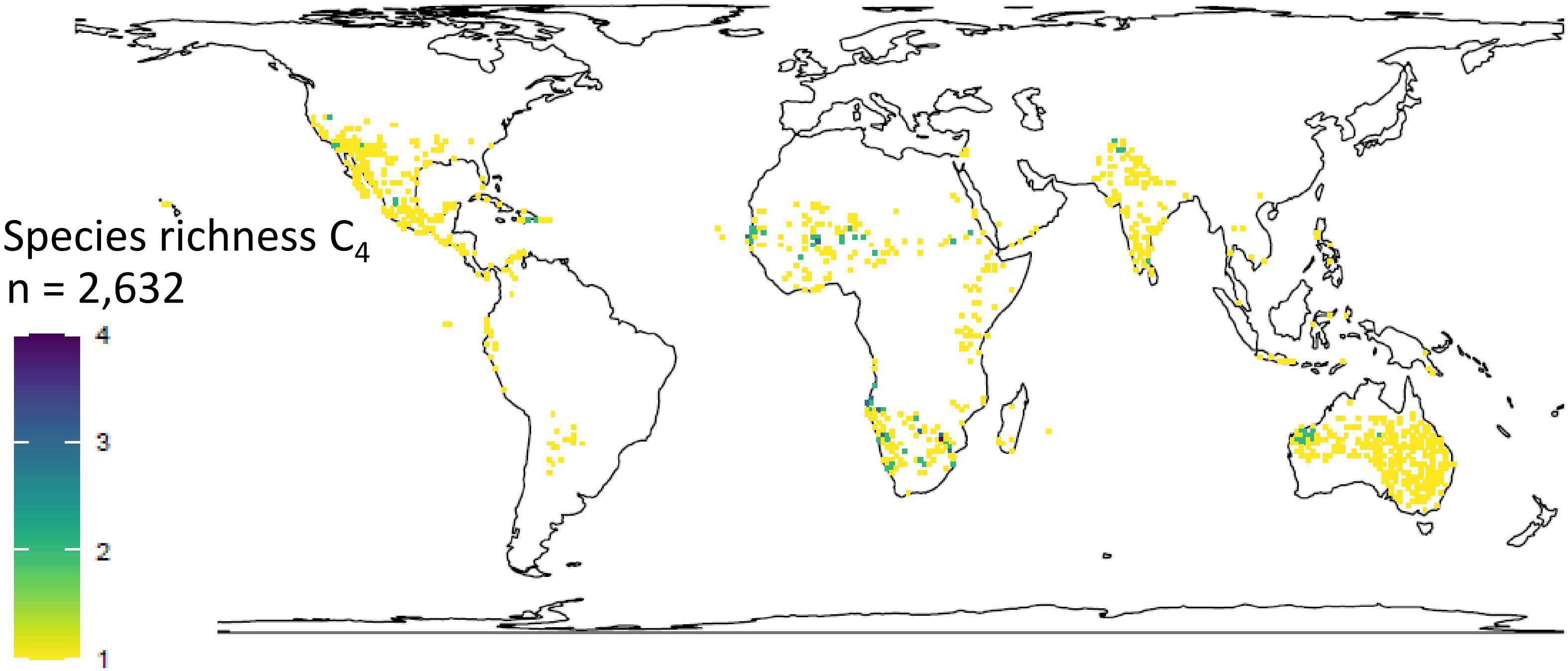

Figure A3

Amaranthaceae

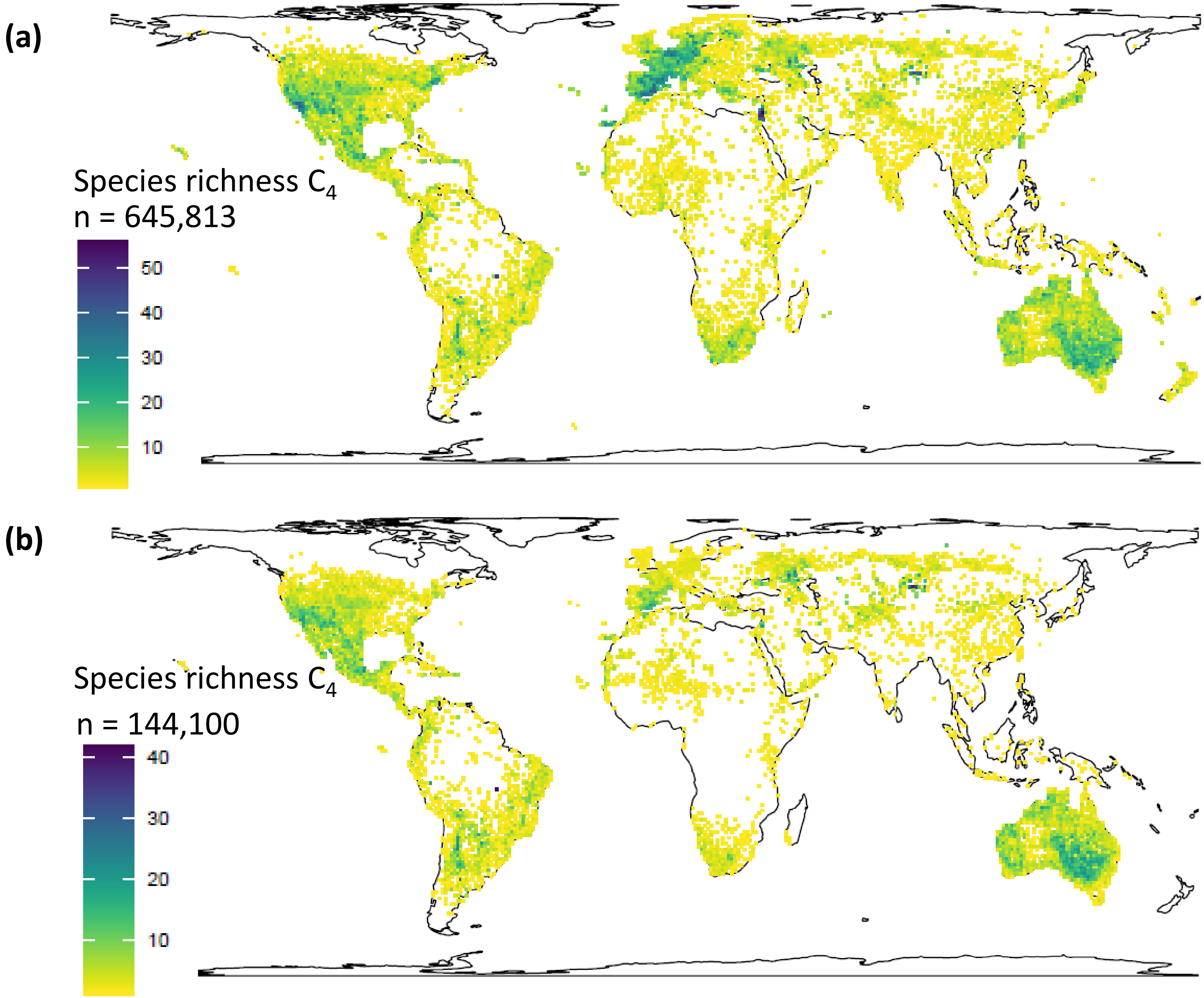

Figure A4

Asteraceae

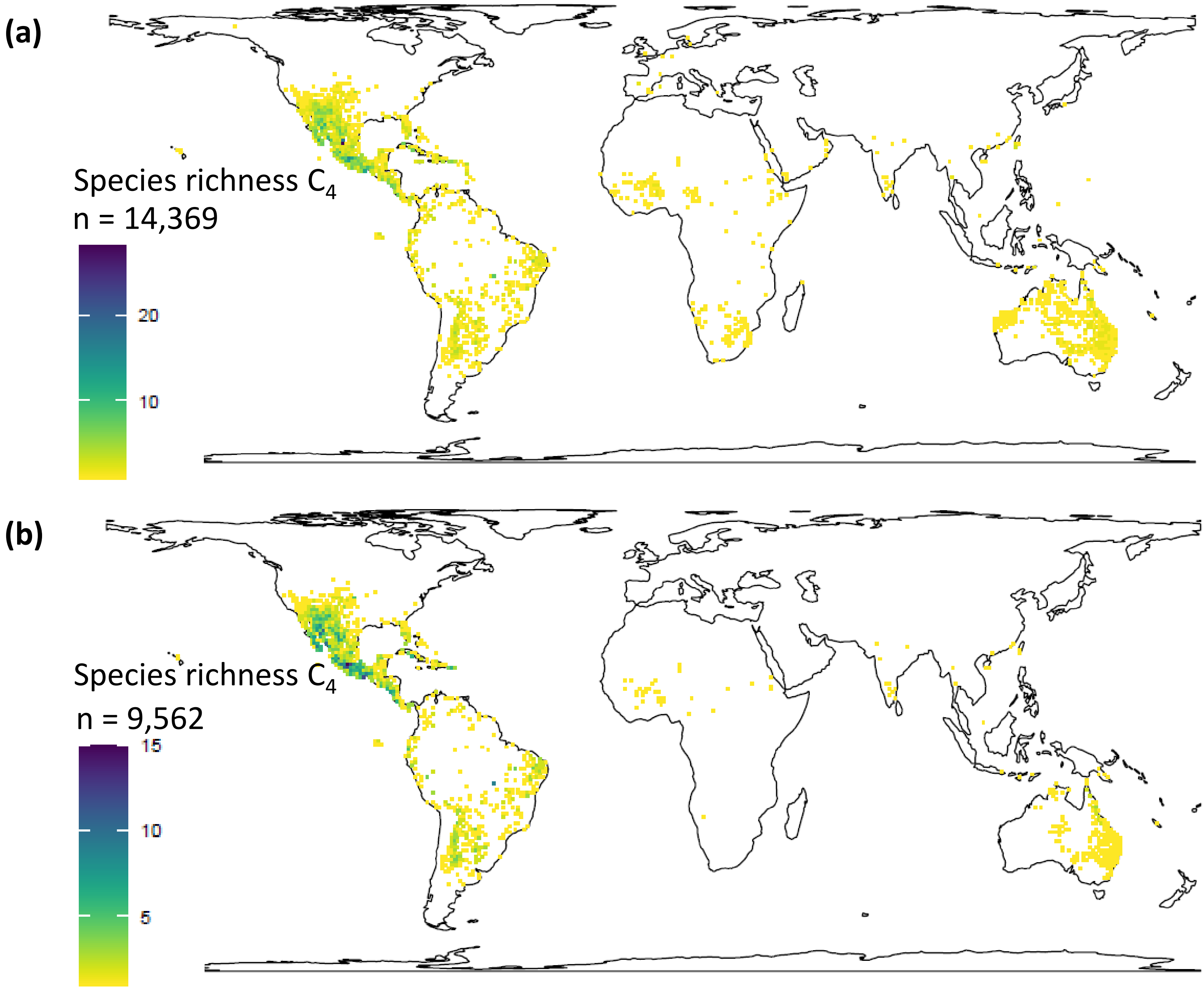

Figure A5

Boraginaceae

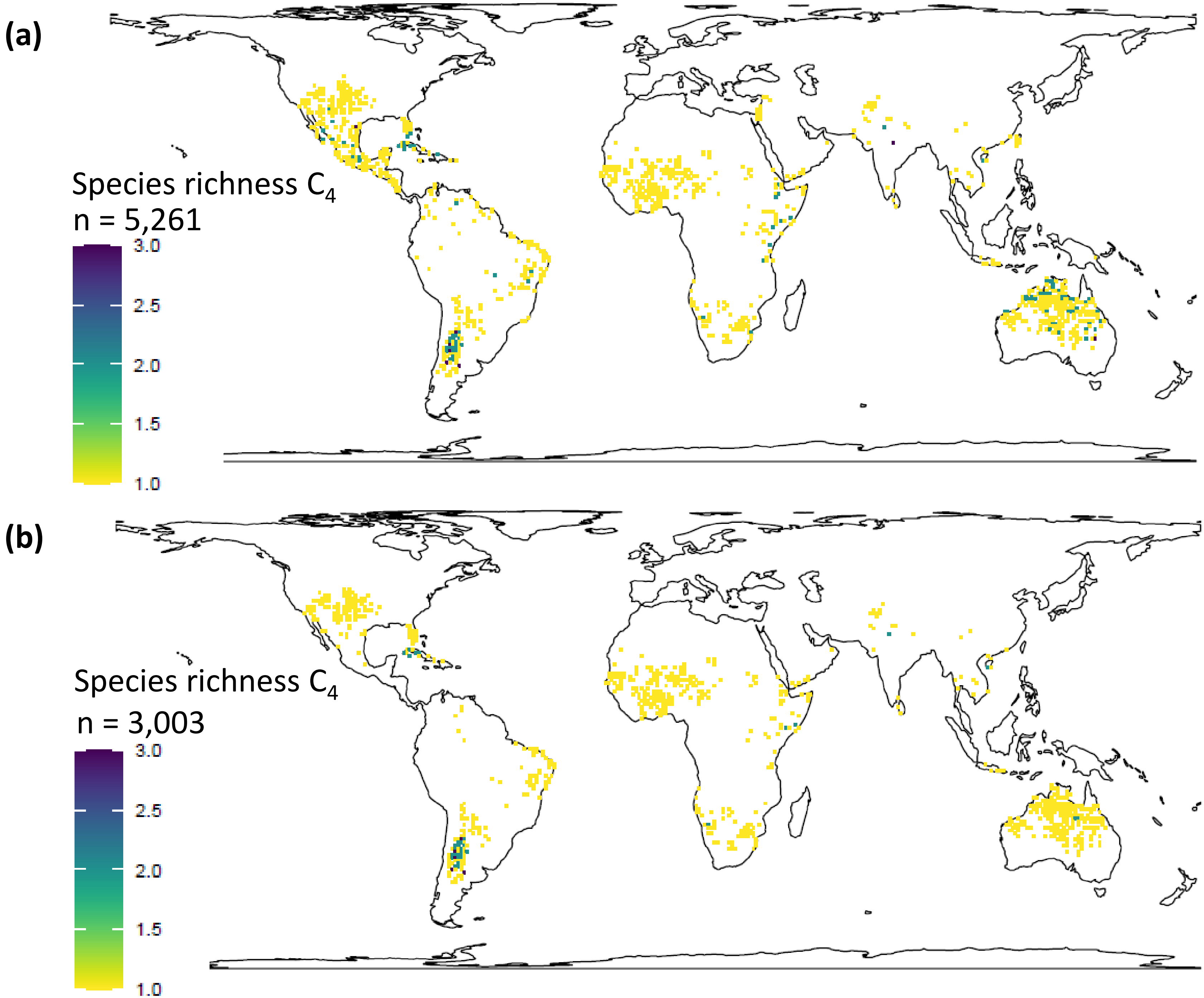

Figure A6

Caryophyllaceae

(a)

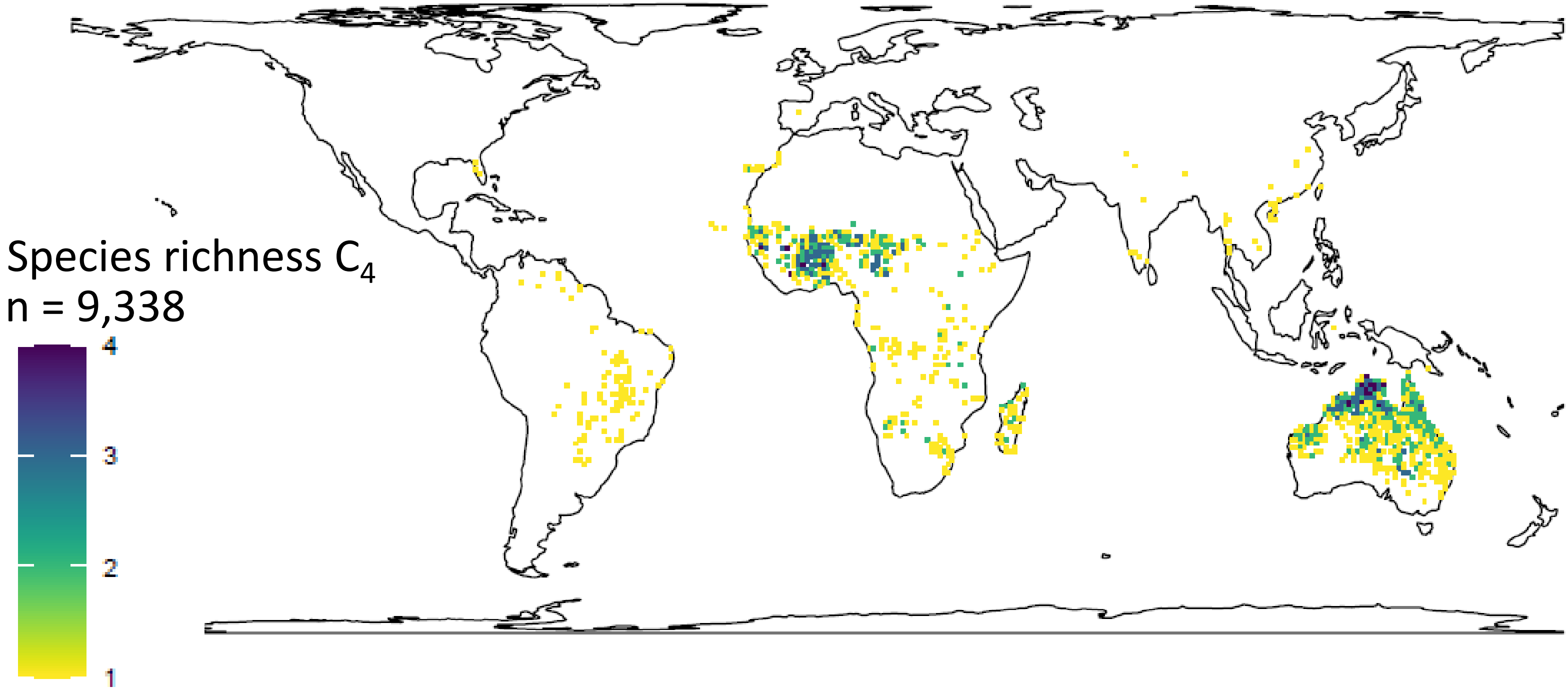

(b)

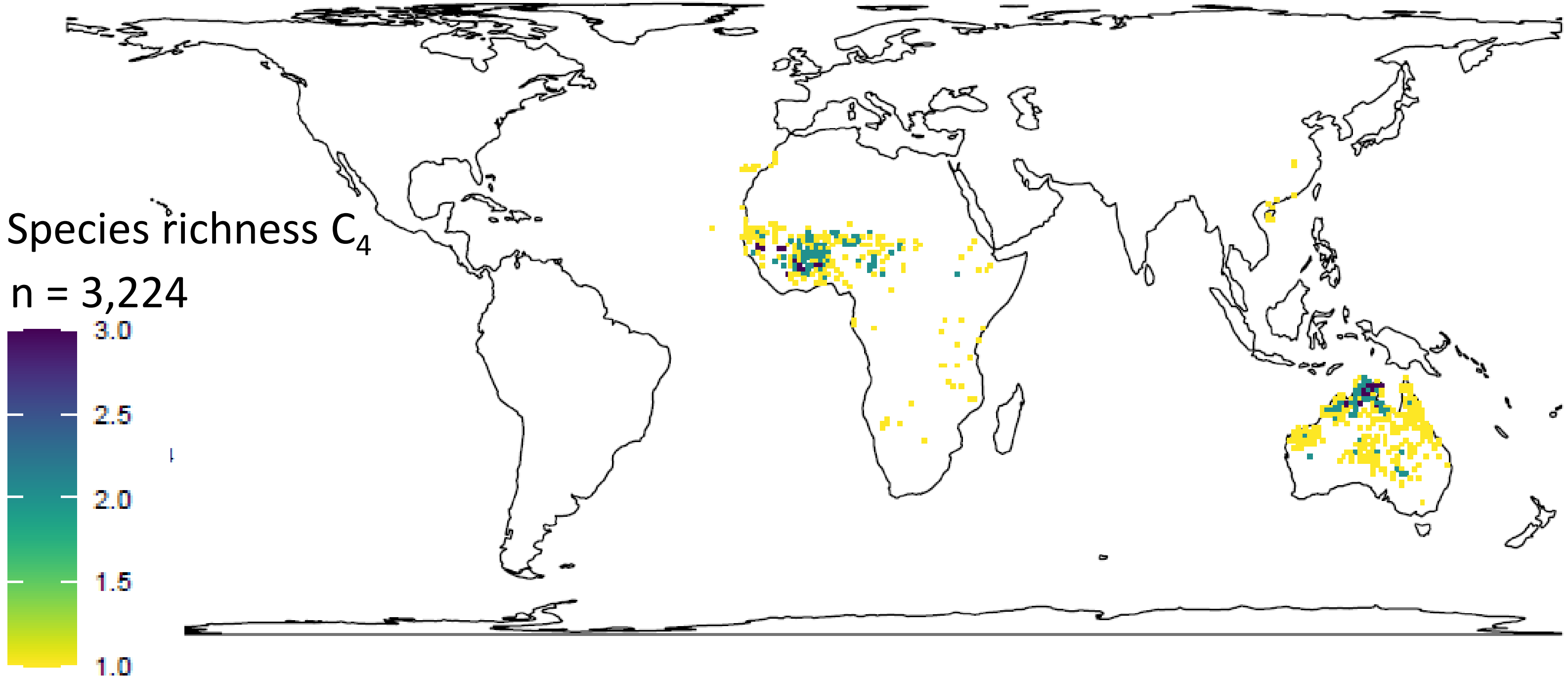

Figure A7
Cleomaceae

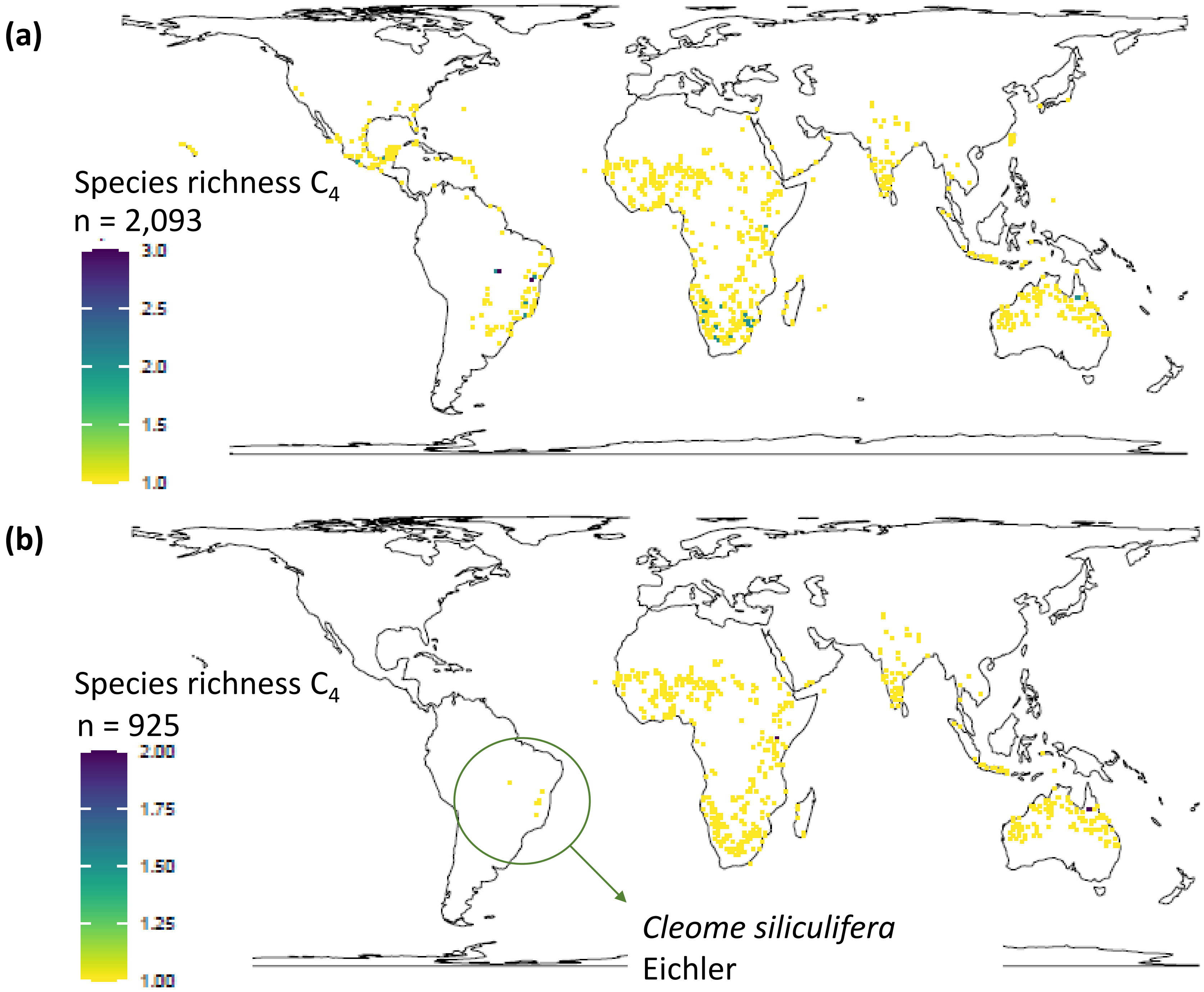

Figure A8

Euphorbiaceae

(a)

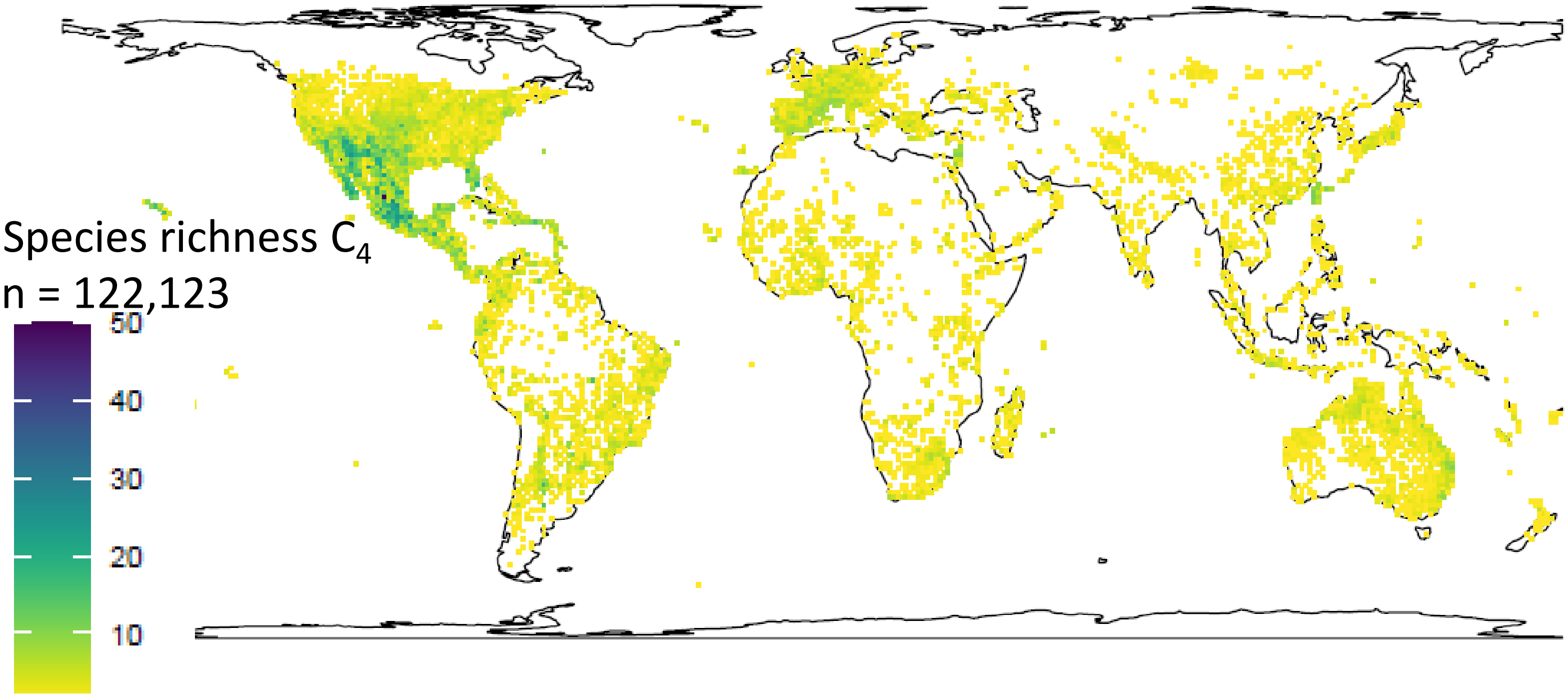

(b)

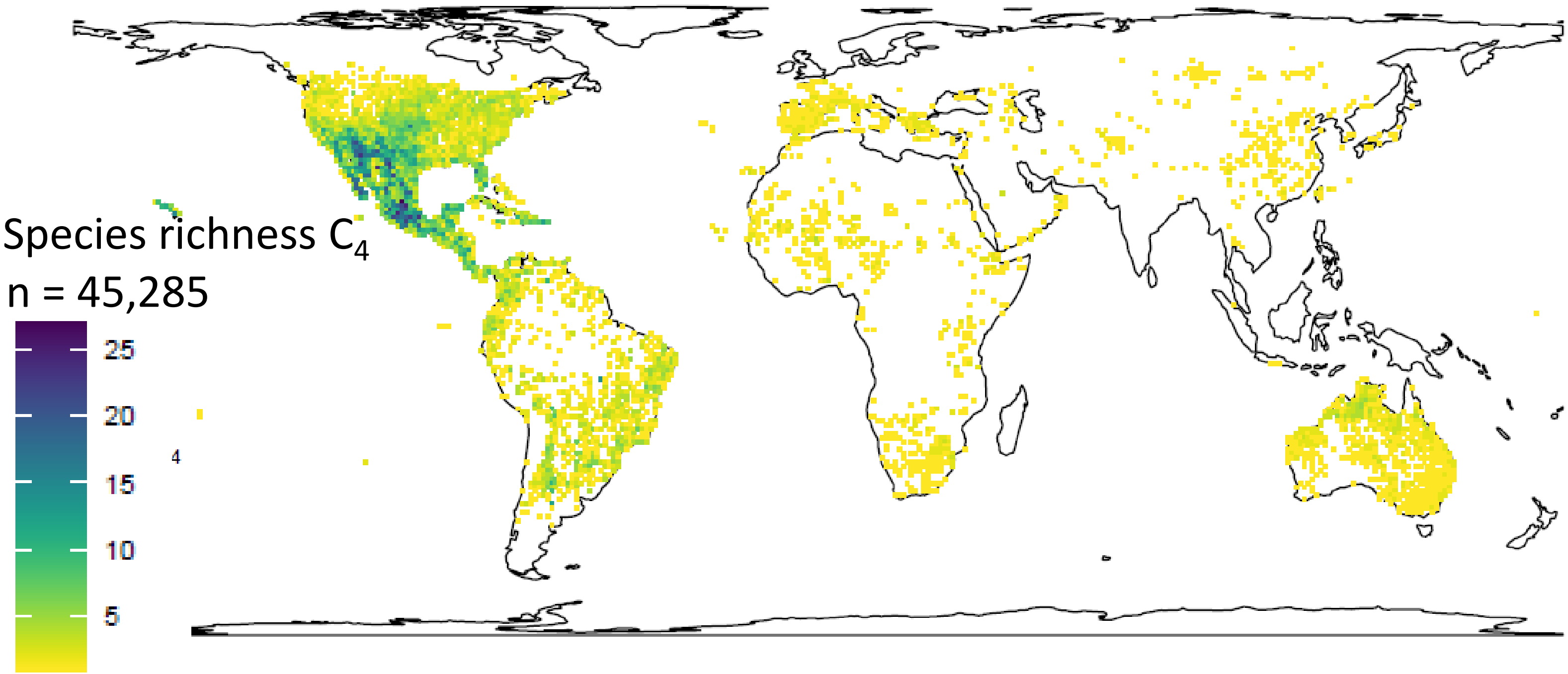

Figure A9

Gisekiaceae

(a)

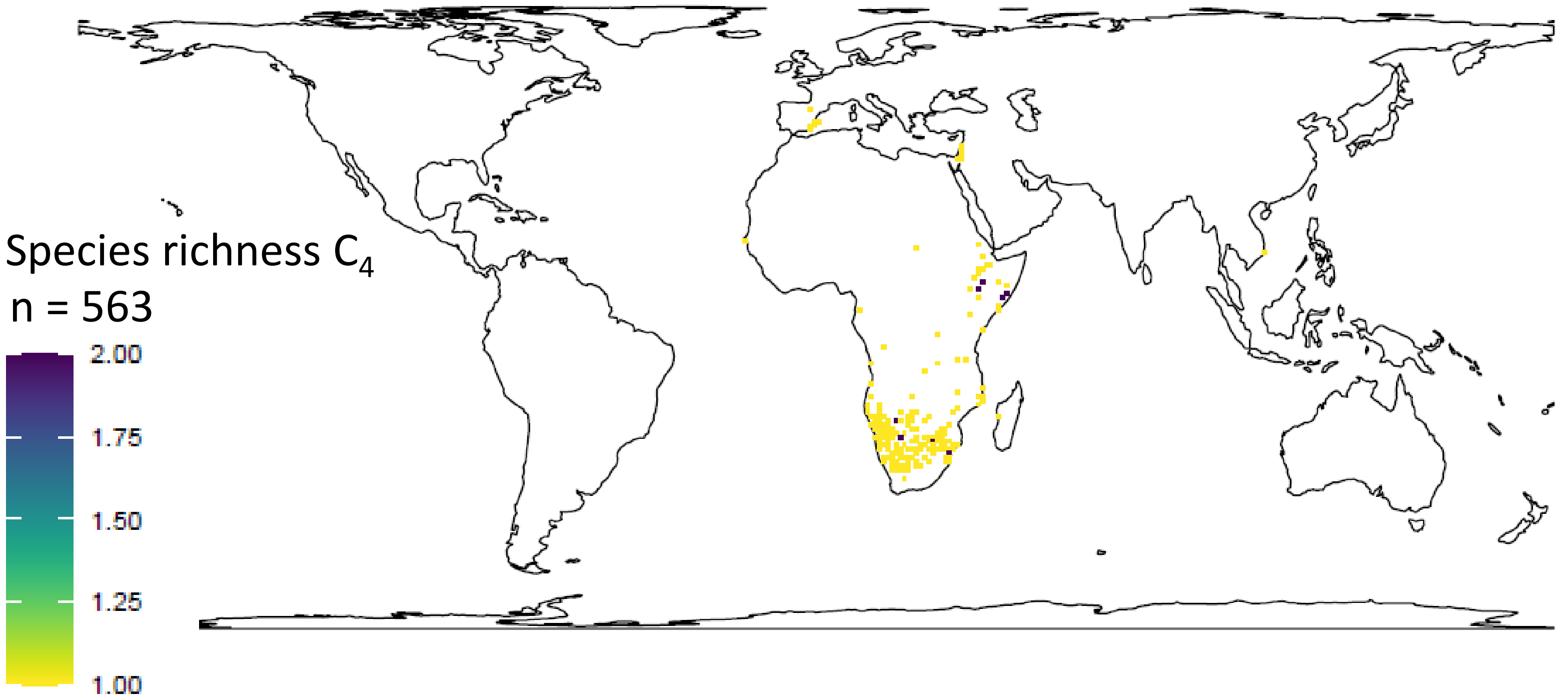

(b)

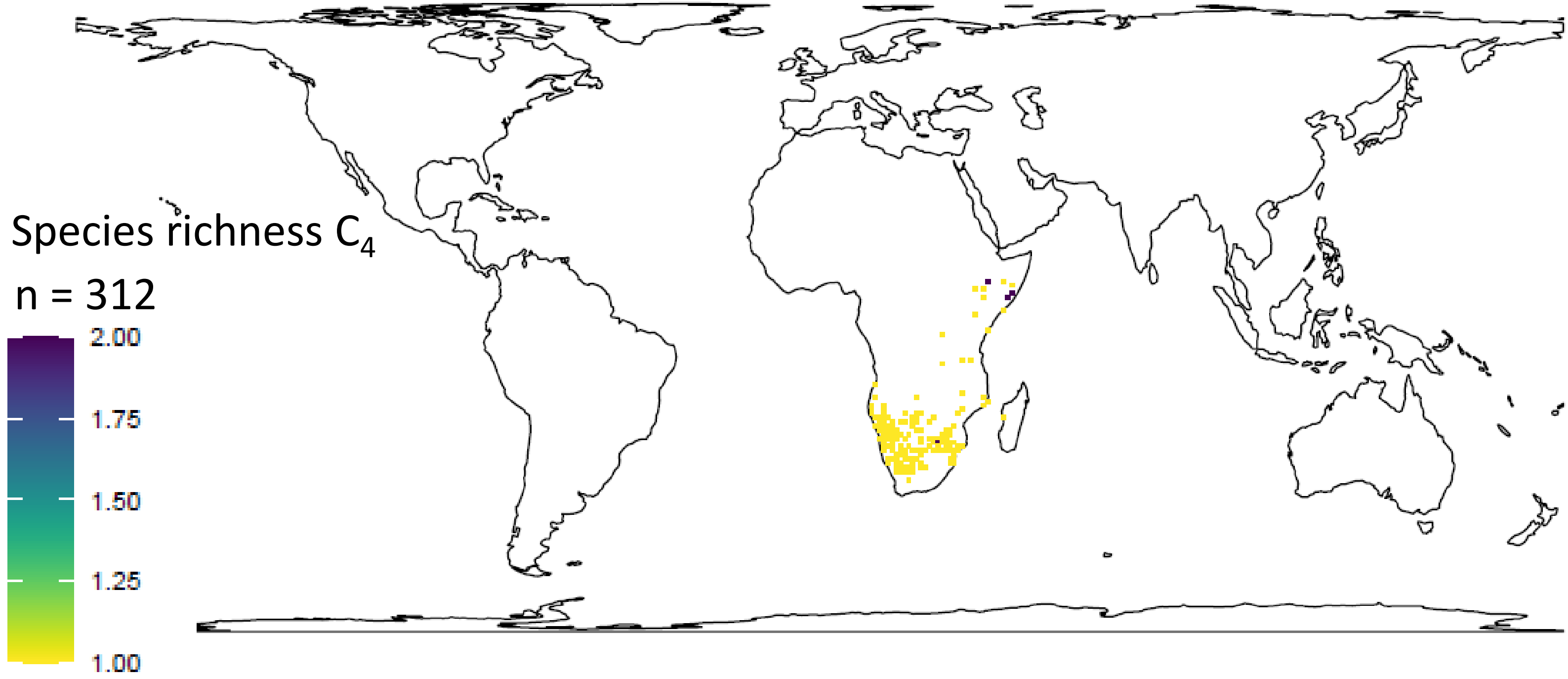

Figure A10

Molluginaceae

(a)

(b)

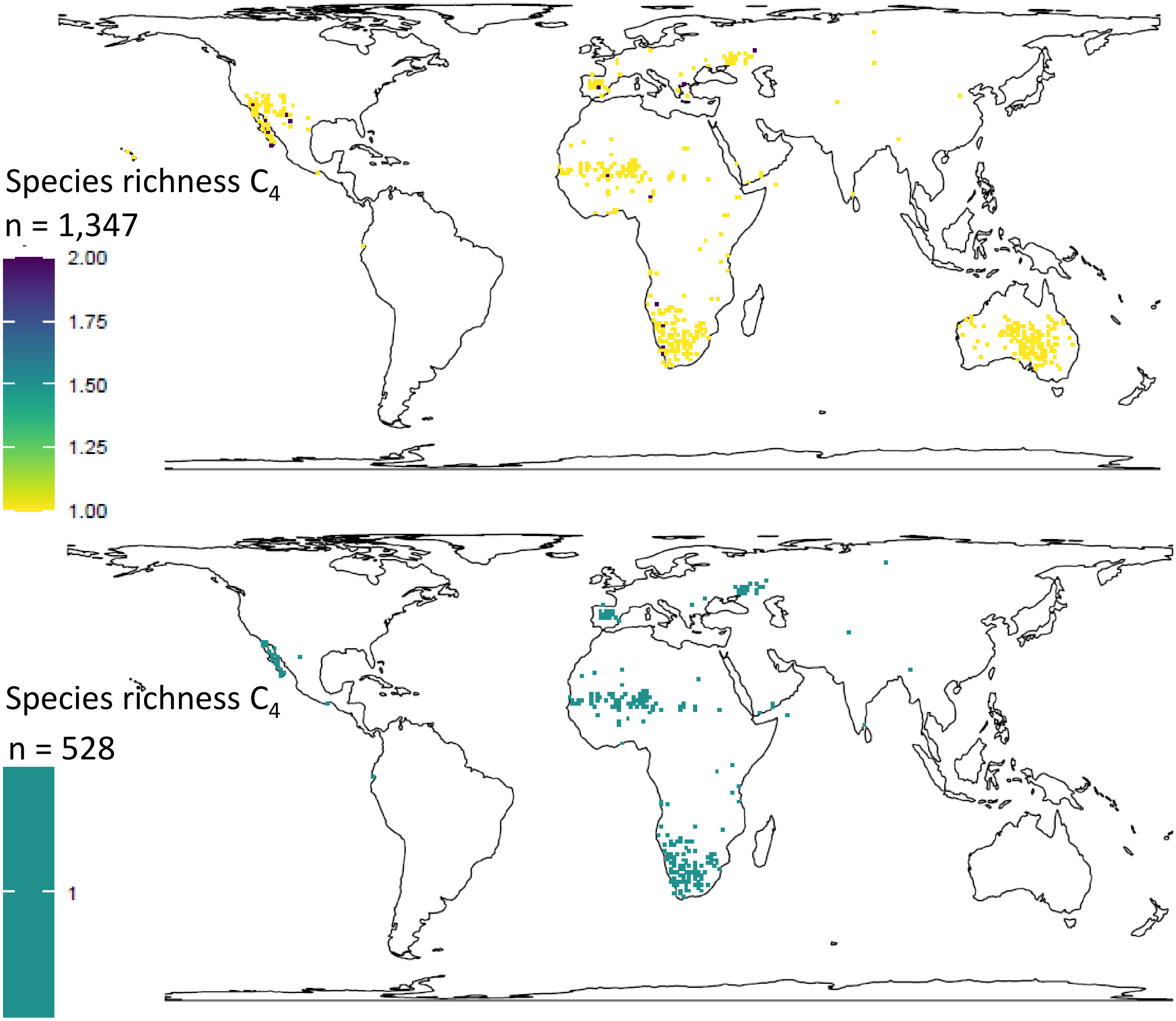

Figure A11

Nyctaginaceae

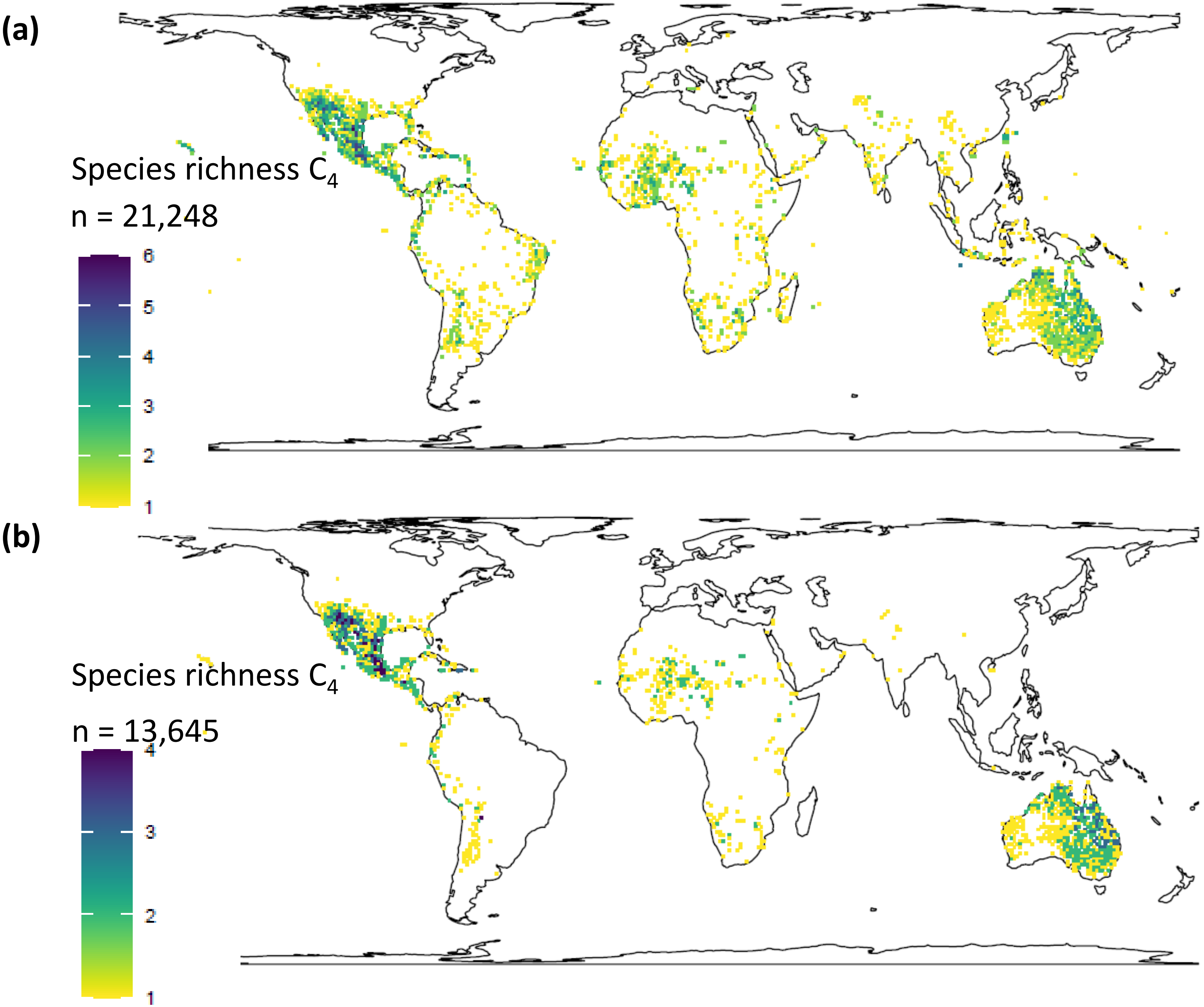

Figure A12

Polygonaceae

(a)

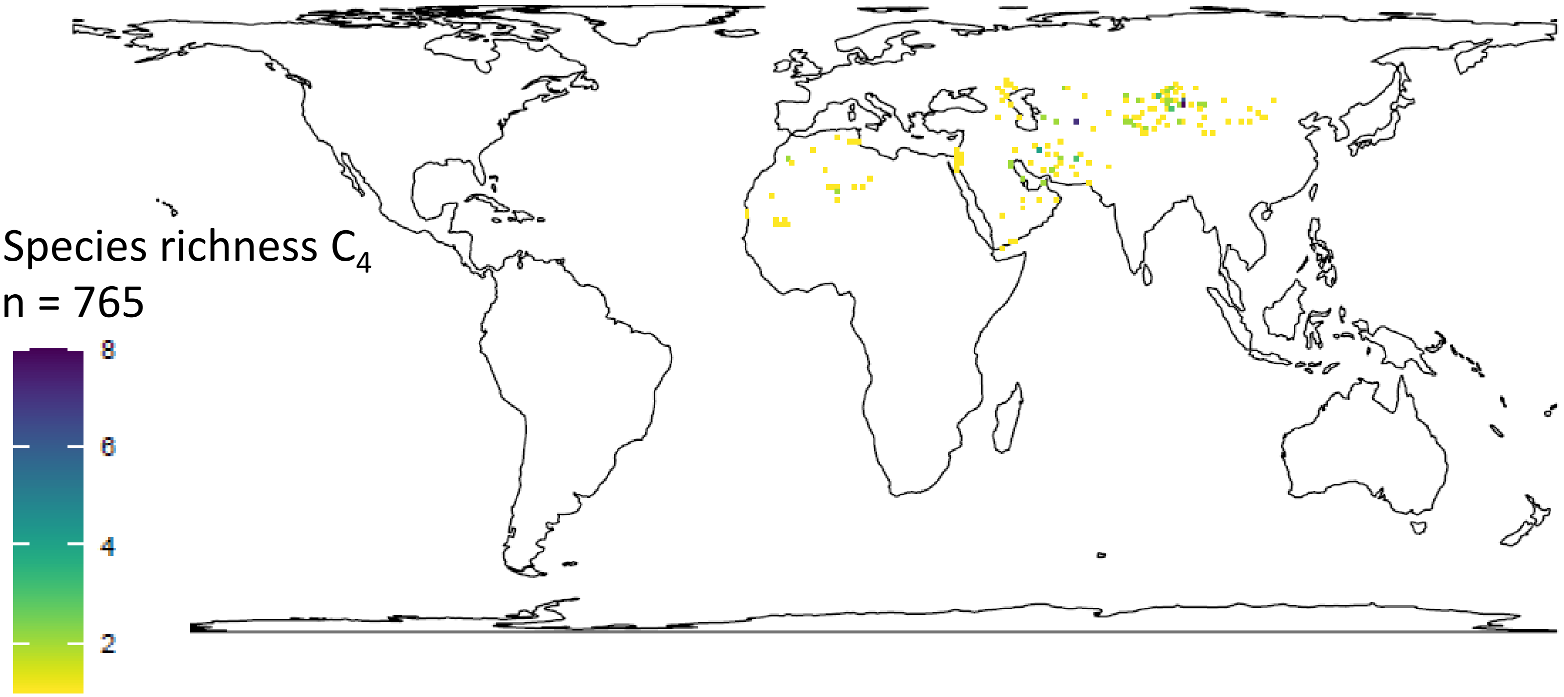

(b)

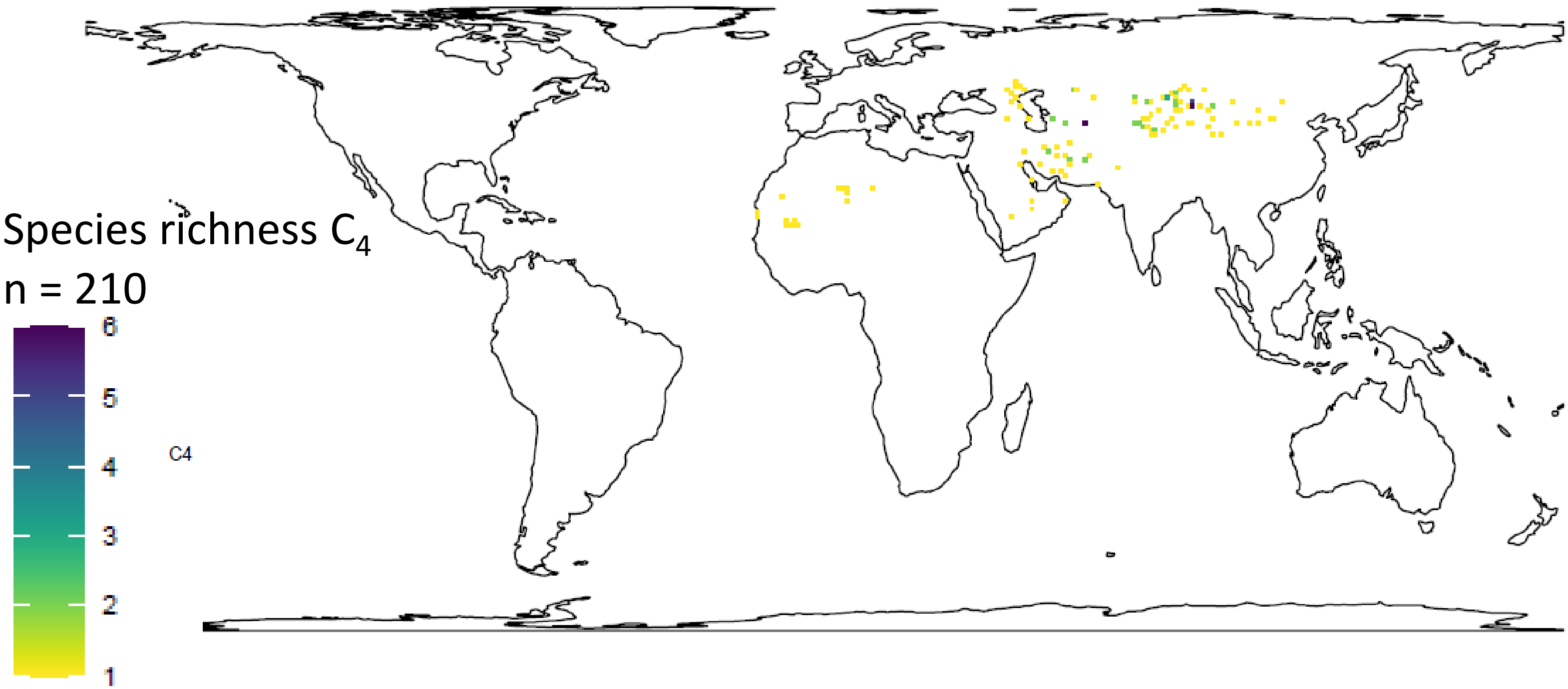

Figure A13

Portulacaceae

(a)

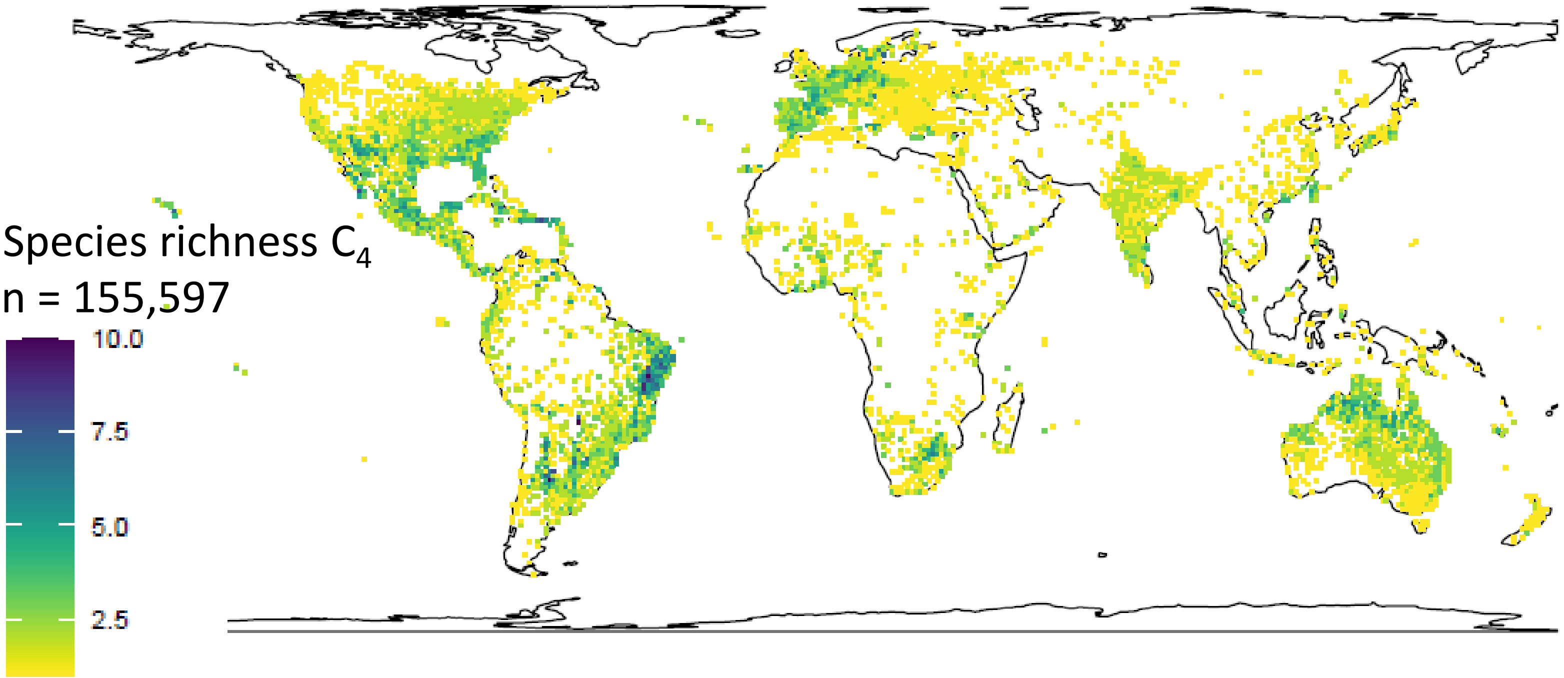

(b)

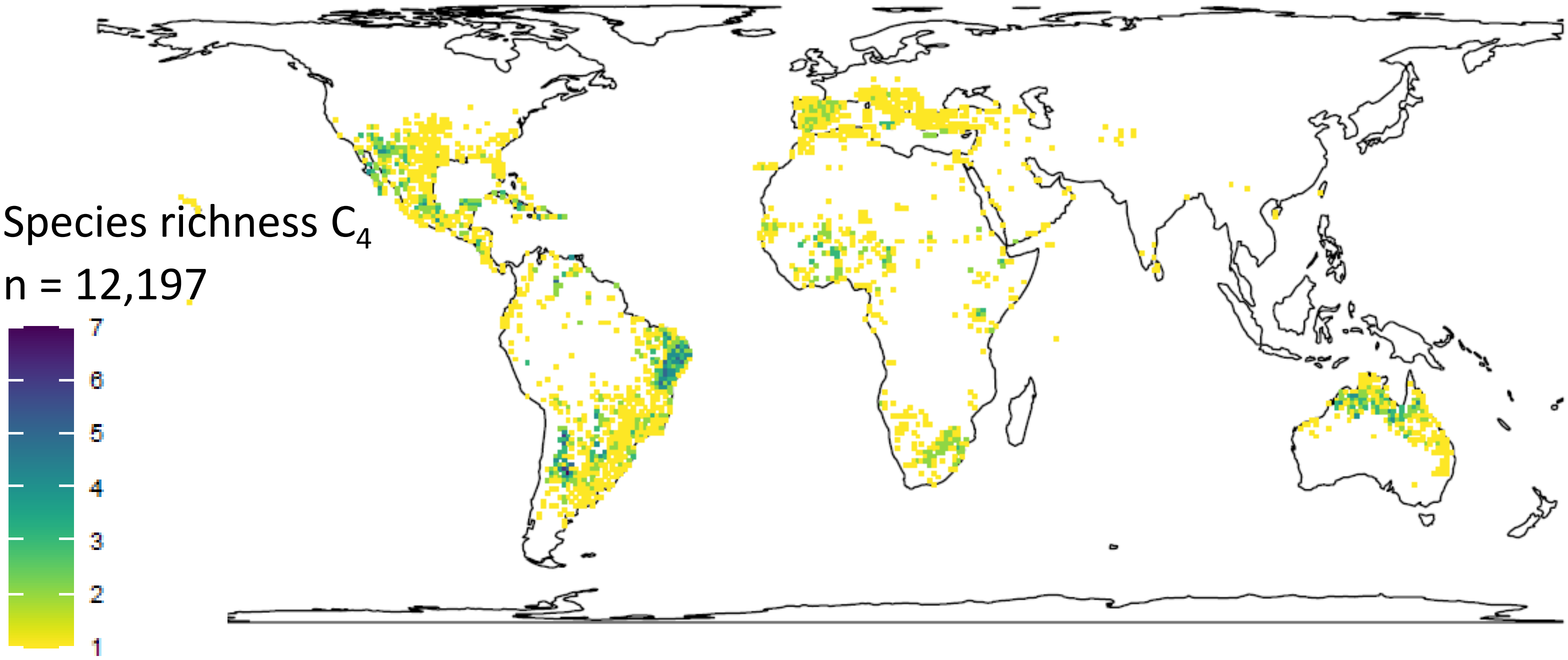

Figure A14

Scrophulariaceae

(a)

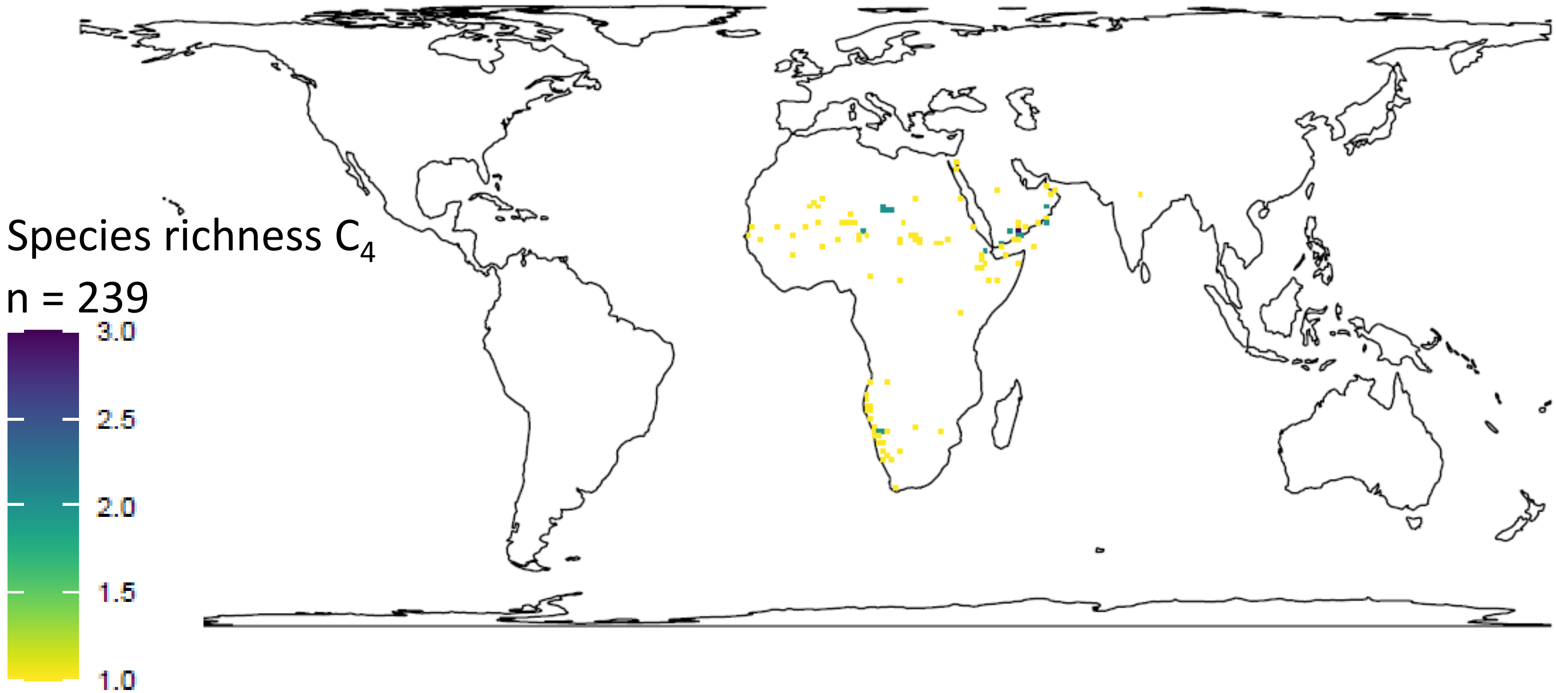

(b)

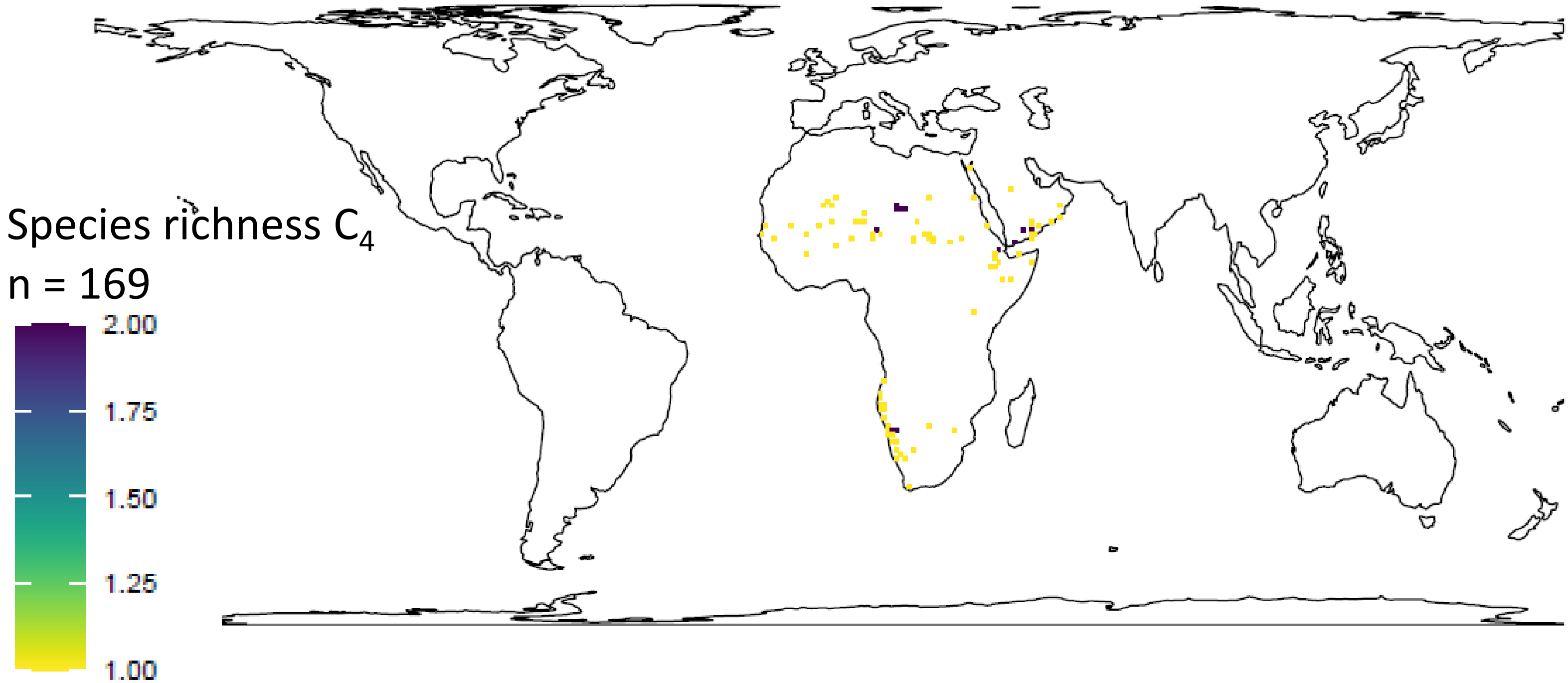

Figure A15

Zygophyllaceae

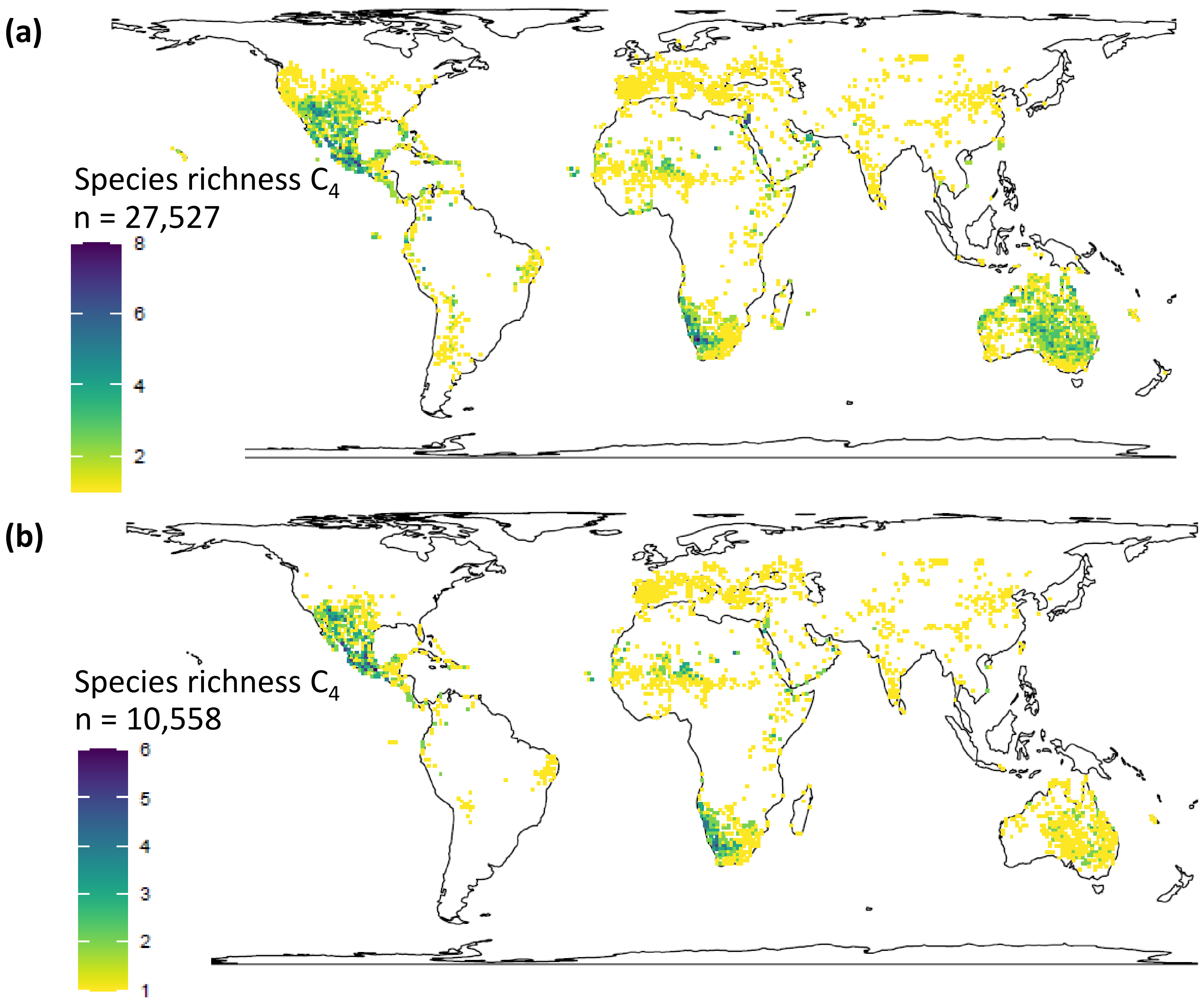

Supplement: Supplementary file 1 — Appendix S1 [file ECE3-13-e10720-s001.zip › ece310720-sup-0001-Figures.pdf]
