# Supplementary material for: Global distribution, climatic preferences and photosynthesis‐related traits of C4 eudicots and how they differ from those of C4 grasses
Source: Ecol Evol. 2023 Nov 12;13(11):e10720. doi: 10.1002/ece3.10720 (PMC10641307; doi:10.1002/ece3.10720)

Figure A16: Scatterplot showing annual mean temperature (°C) and annual mean precipitation (mm) data of C_4 grasses (green) and C_4 eudicot (blue) occurrence points per continent.

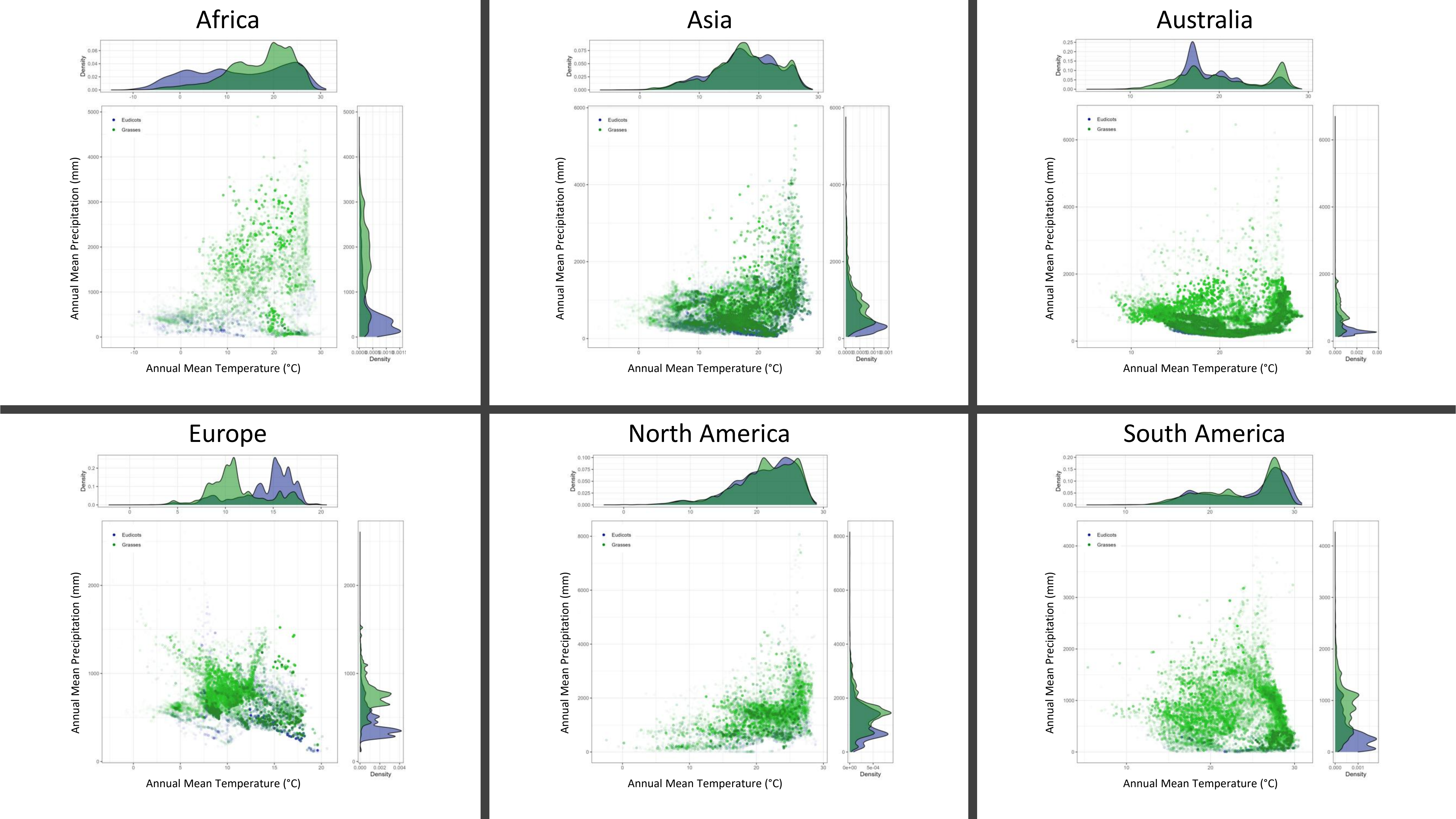
Africa
Asia
Australia
Europe
North America
South America
Density
Eudicots
Grasses
Annual Mean Precipitation (mm)
Annual Mean Temperature (°C)

Africa

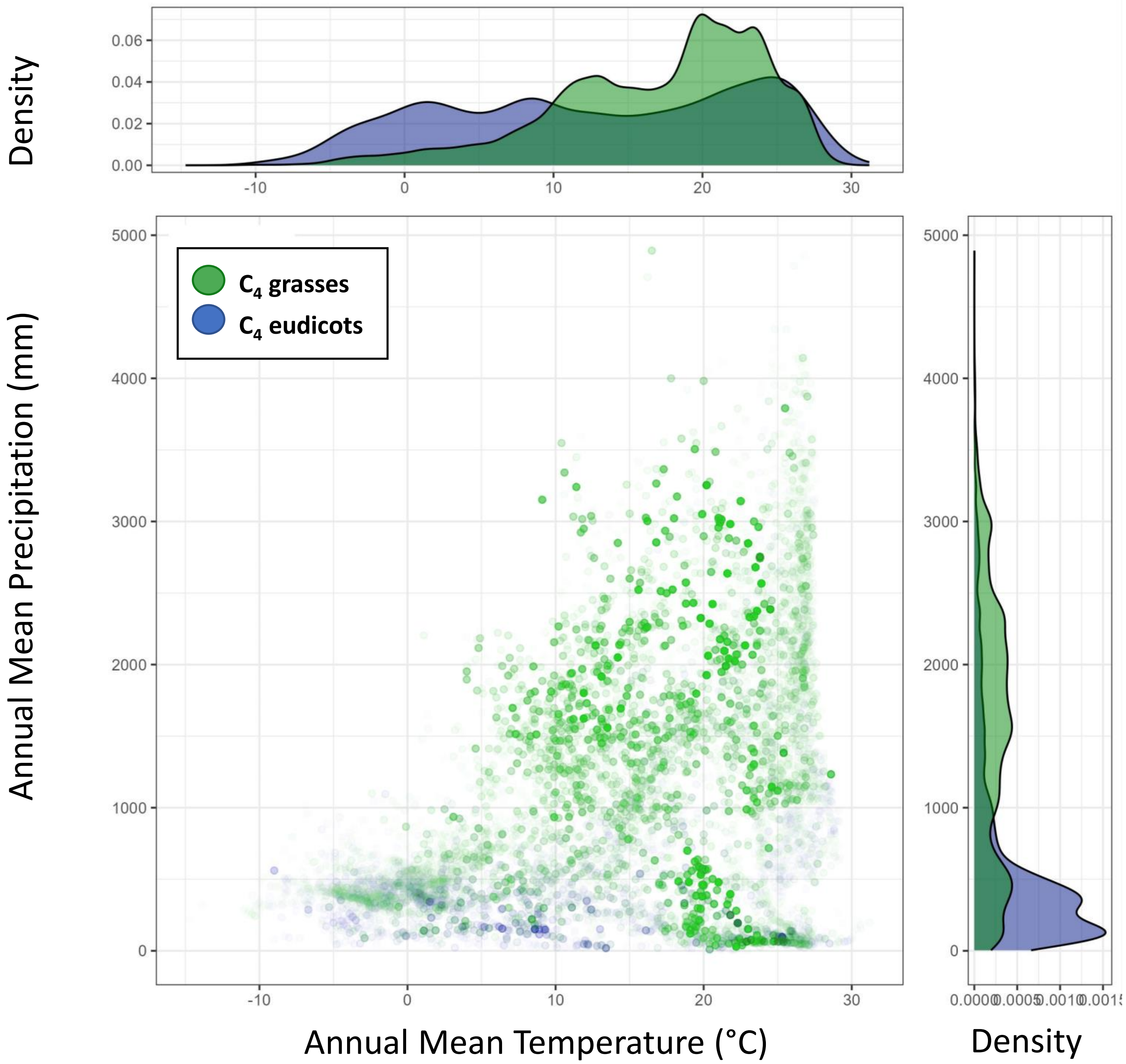

Density
0.06
0.04
0.02
0.00
-10
0
10
20
30
Annual Mean Precipitation (mm)
5000
4000
3000
2000
1000
0
C_4 grasses
C_4 eudicots
Annual Mean Temperature (°C)
Density

Asia

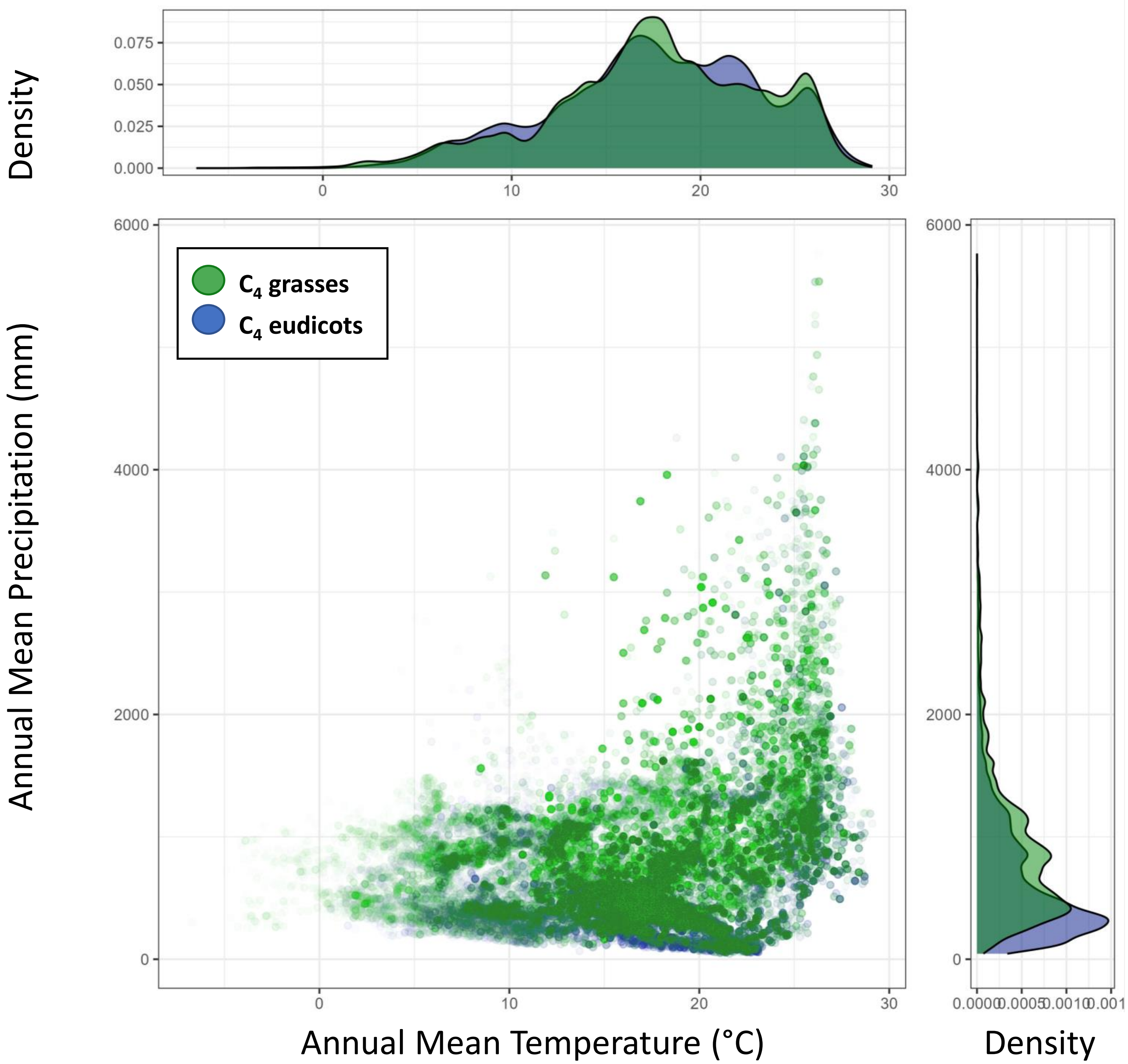
Density
0.075
0.050
0.025
0.000
0
10
20
30
6000
4000
2000
0
C4 grasses
C4 eudicots
Annual Mean Precipitation (mm)
Annual Mean Temperature (°C)
0.0000
0.0005
0.0010
0.001
Density

Australia

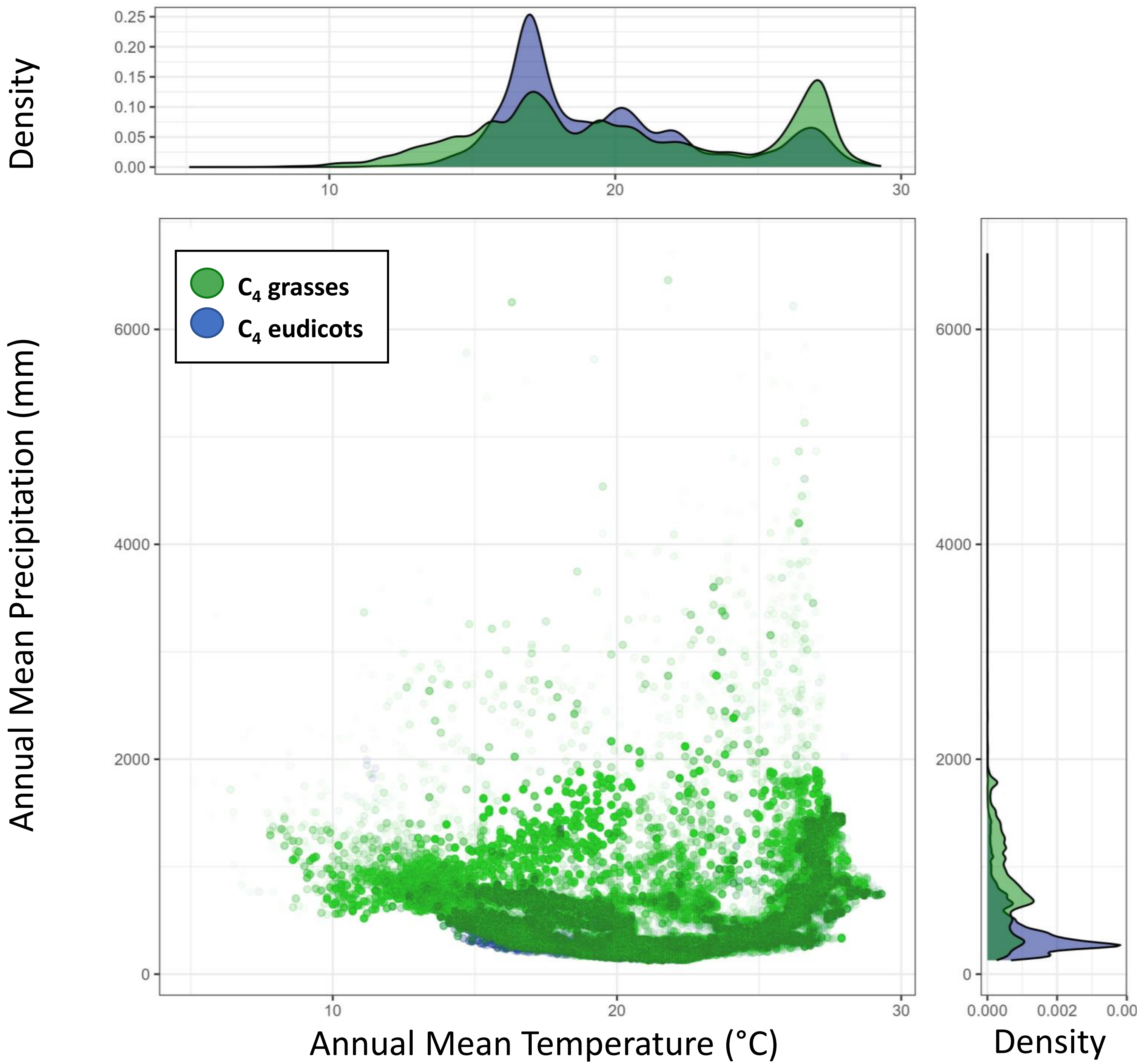

Density
0.25
0.20
0.15
0.10
0.05
0.00
10
20
30
C4 grasses
C4 eudicots
Annual Mean Precipitation (mm)
6000
4000
2000
0
Annual Mean Temperature (°C)
0.000
0.002
0.00
Density

Europe

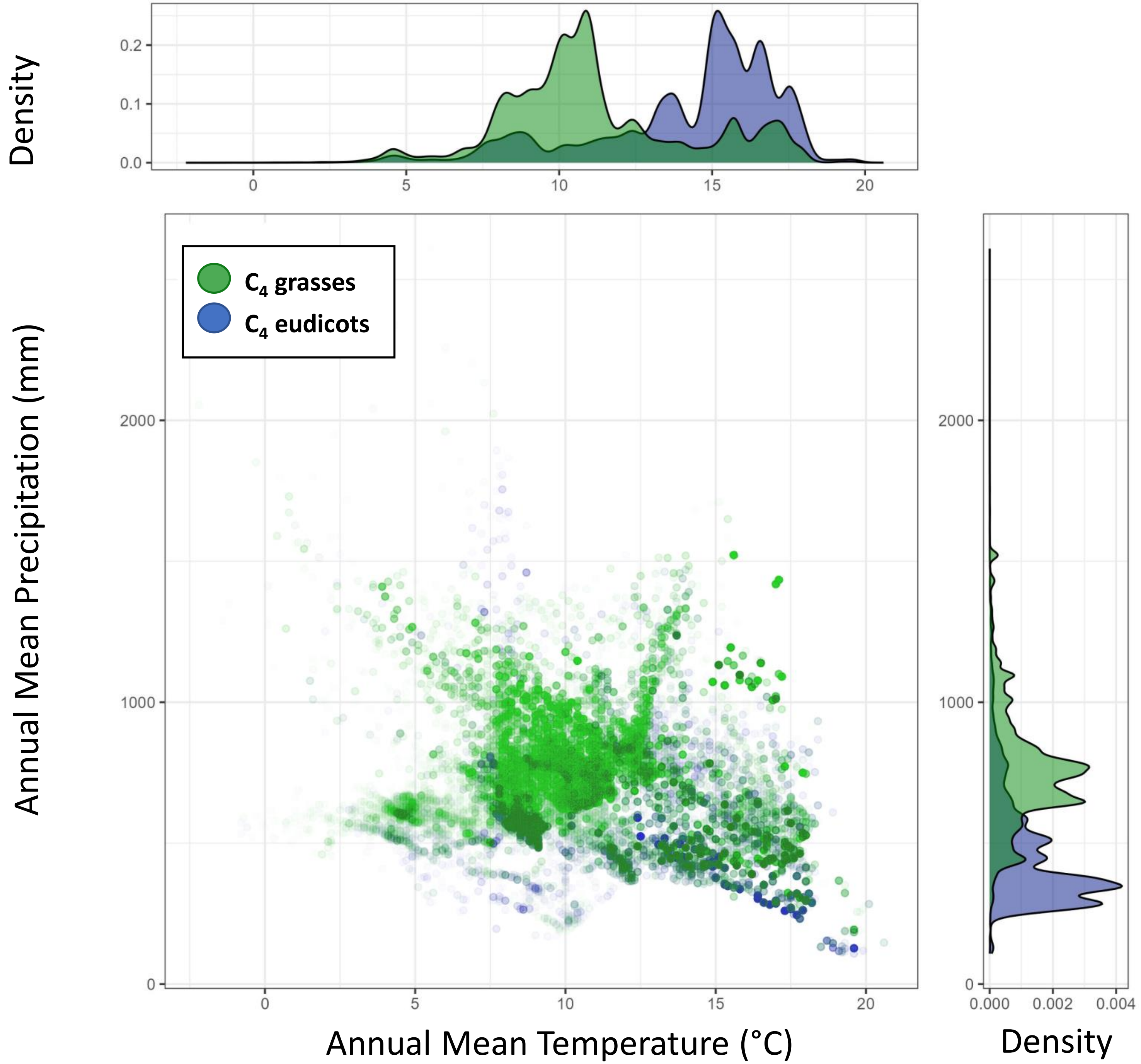
Density
0.2
0.1
0.0
0
5
10
15
20
C4 grasses
C4 eudicots
Annual Mean Precipitation (mm)
2000
1000
0
Annual Mean Temperature (°C)
0.000
0.002
0.004
Density

North America

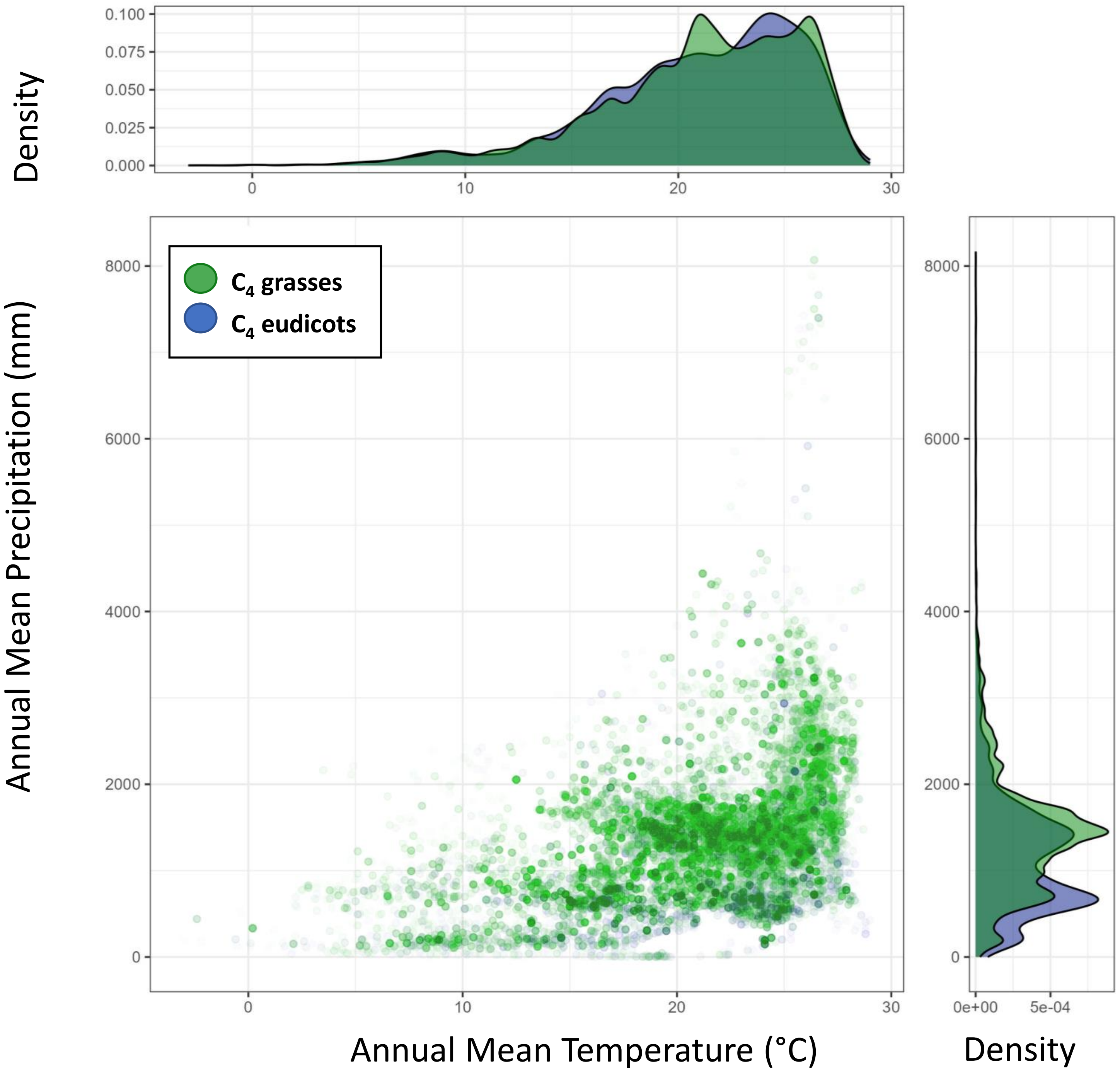

South America

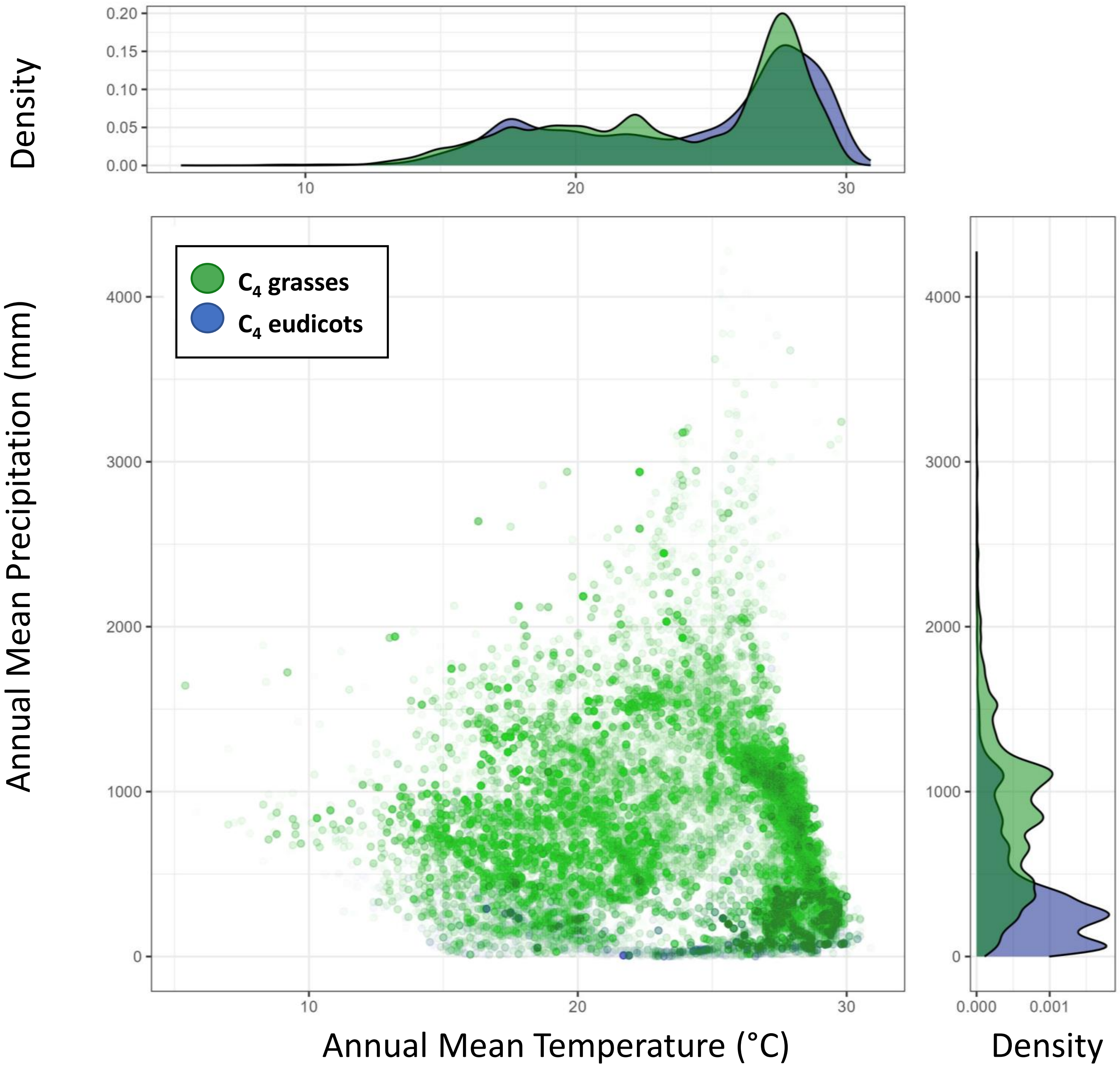

Supplement: Supplementary file 1 — Appendix S1 [file ECE3-13-e10720-s001.zip › ece310720-sup-0002-FigureA16.pdf]
